# Relevance and challenges of exposome studies for environmental health research

**DOI:** 10.1186/s12940-026-01299-3

**Published:** 2026-05-29

**Authors:** Rémy Slama, Valérie Siroux, Martine Vrijheid, Xavier Basagaña

**Affiliations:** 1 SMILE Team, Institut de Biologie de L’ENS (IBENS), Ecole Normale Supérieure, Université PSL, CNRS, 75005 Paris, Inserm France; 2 PARSEC (Paris Research in Health, Environment and Climate), Ecole Normale Supérieure, Inserm, 75005 Paris, France; 3 UMR 5309, Team of Environmental Epidemiology Applied to Development and Respiratory Health, Institute for Advanced Biosciences, University Grenoble Alpes, CNRS, Inserm U 1209, Grenoble, France; 4 ISGlobal, Barcelona, Spain; 5 Universitat Pompeu Fabra, Barcelona, Spain; 6 Spanish Consortium for Research On Epidemiology and Public Health (CIBERESP), Madrid, Spain

## Abstract

The exposome encompasses all environmental exposures humans undergo from conception. We frame exposome studies according to four broad aims essential for environmental health research. First, a descriptive aim, consisting in assessing exposure patterns, including correlations between exposures and their within-subject variability. This descriptive aim includes “environmental justice” studies of associations between the exposome and sociodemographic factors. A second – etiologic – aim is to describe the subclinical and clinical effects of the exposome (hazard identification). A third aim is the quantification of the exposome health impact (e.g., in life years lost at the population level) and the ranking of exposures in terms of population disease burden (exposome disease burden or risk assessment). A fourth (intervention) aim corresponds to the identification of approaches to modify the exposome, as a way to improve health. With the large increase in the number of chemicals quantifiable in a small volume of a biospecimen, the main challenge of descriptive exposome studies is population representativeness. Regarding the etiologic aim, by simultaneously showing associations of a given biological parameter with hundreds of exposures, exposome studies effectively limit publication bias and selective reporting of results, which are a strong concern in single-exposure studies. They nonetheless face several challenges, related to the curse of dimensionality, the correlation between exposures, the breadth vs. depth tension… Increasing the number of exposures considered implies to simultaneously increase study population sizes, aiming for cohorts of 100,000 subjects or more. The accuracy of exposure assessment should simultaneously be improved, e.g., by collecting repeated biospecimens within each subject, assessing exposures at various ages and decreasing limits of quantification. Classical exposome statistical designs such as ExWAS (exposome-wide association studies) are subject to a high false positive rate. Models adapted to sparse data allowing to control for confounding by co-exposures appear more efficient. The results from exposome descriptive and etiologic studies can be combined to feed exposome disease burden assessments. These can in turn help prioritize exposures for which efficient interventions need to be identified. The approaches outlined in this work could help exposome research contribute more strongly to environmental health research and to the associated risk management decisions.

**Supplementary Information:**

The online version contains supplementary material available at 10.1186/s12940-026-01299-3.

## Introduction

Environmental health research can be defined as the study of the influence of factors of external origin on human health. It has key major goals including: to describe human exposures and identify positive or negative determinants of health-related states; to understand the (biological, sociological) mechanisms underlying the effects of these factors; to quantify the corresponding population impact (e.g., as an attributable number of disease cases or disability adjusted life years, or DALYs) and finally to identify ways to modify this impact in order to improve population health. In humans, and to some extent in animal studies, these questions are currently mainly tackled via studies considering a single exposure, or exposure family, at a time (“single exposure studies”).

Single exposure studies have clear merits, such as the ability to go to some extent “in depth”, but also several recognized limitations. These limitations include, when it comes to the identification of effects of environmental exposures: the potential for selective reporting of associations, hidden multiple testing (sometimes termed HARKing, for Hypothesizing After the Results are Known) and publication bias [1]; the difficulty to control for co-exposures acting as potential confounders; challenges to address potential “mixture” effects, including synergy between environmental exposures; difficulties in comparing or ranking exposures based on the magnitude of their health effect or health impact; a rather low throughput, in terms of the number of exposures considered per researcher and per year or per article (Table 1). One important question is whether exposome studies could overcome some of these limitations.

**Table 1:** Some challenges of “single exposure” studies, with indications on whether exposome studies could allow to overcome them

Issue	Addressed by exposome studies?
*Confounding*	
Confounding by co-exposures	Yes possibly, depending on the analytical approach (e.g., not addressed in an ExWAS analysis) [2] and assuming that the relevant exposures have been assessed
Confounding by other factors	Not necessarily better addressed in exposome studies (possibly more poorly if factors requiring to be controlled for are not selected on an exposure-by-exposure basis)
*Selection bias*	
Selective reporting of associations	Yes if exposome studies report all tested associations (e.g. in appendix)
Publication bias [1]	Yes, in principle if exposome studies report all associations and are all accessible
Selection bias in the study population [3]	No. On the contrary, given a generally increased participation burden, exposome studies may be more prone to selection bias than true single exposure studies
*Measurement error*	
In exposures	May be worse in exposome studies if the increase in the number of exposures considered is done at the cost of a decrease in the accuracy of the assessment of each individual exposure
In outcome and confounders	Possibly worsened situation for confounders if the increase in the number of confounders assessed entails a decrease in the quality of the assessment of confounders or of their selection. No strong specificity of exposome studies regarding the health outcome
*Effect measure modification*	
Lack of consideration of mixtures effects/synergy	Synergy can be considered in exposome studies but sensitivity and power to detect them may be low [4] and the common challenges in inferring biological interactions from statistical interactions persist [5]
*Issues related to statistical dimension*	
Low throughput (given the number of exposures to test)	Yes, since all associations are published in a single paper
Hidden multiple testing	Yes, since exposures are not considered in distinct papers
Lack of correction for multiple testing	Yes, if desired, although alternative analytical approaches may be more efficient (see text)
Low statistical power	No. Depending on the design and statistical analysis used, power could be lower in exposome studies, unless specific efforts are made (e.g., in terms of increased population size)

**Fig. 1:**
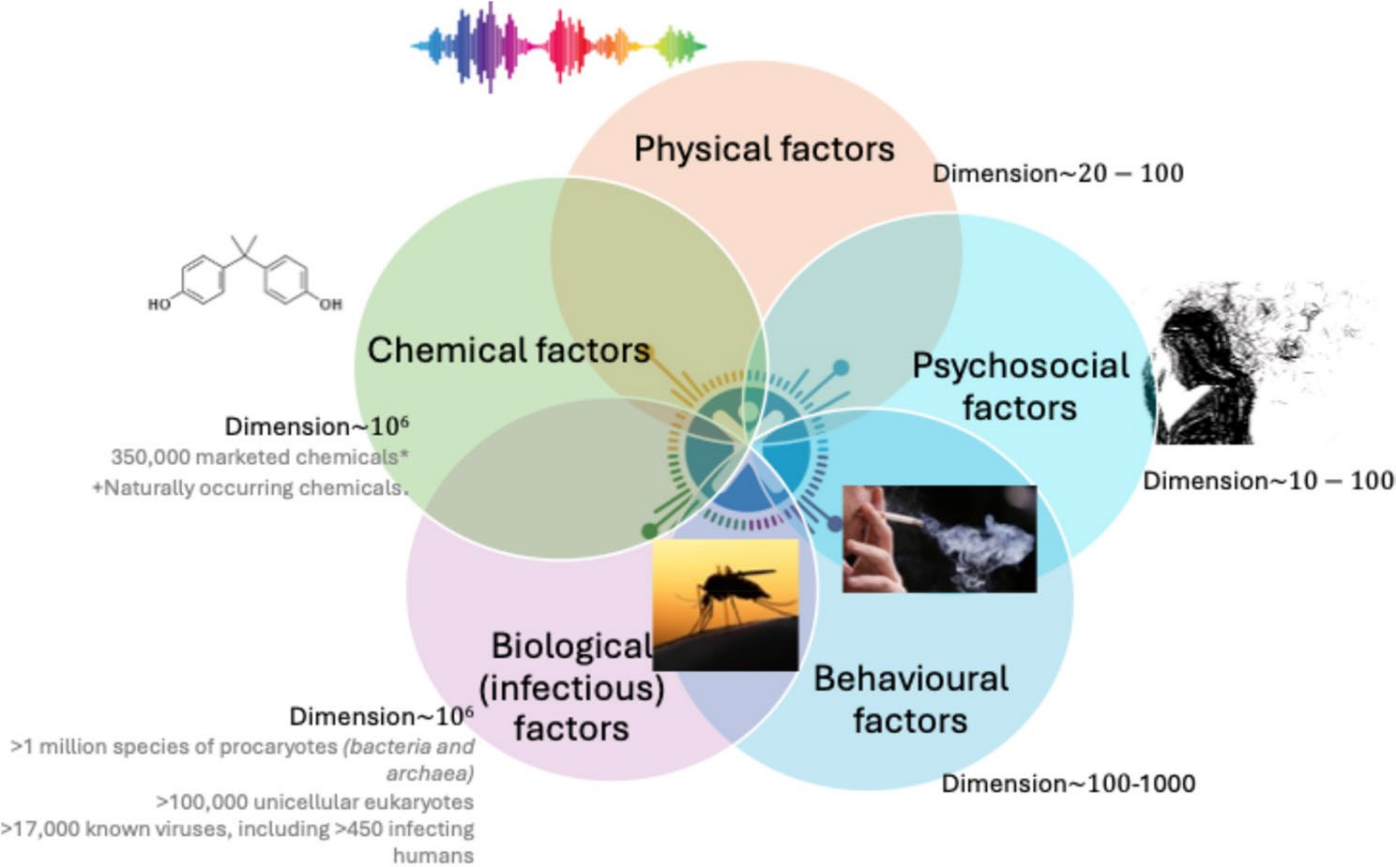
The exposome five main domains. These dimensions can be seen as encompassing lifestyle factors, aesthetic factors and systemic factors such as climate change, antimicrobial resistance or biodiversity loss (see discussion). The approximative number of exposures in each exposome domain (dimension) comes from Wang [11] for chemicals; from Louca for bacteria and archaea [12]; from Wiens for unicellular eukaryotes (considered to correspond to the number of protist species) [13]; from He for virus [14]. See Table 3 for the remaining domains

The exposome concept was defined in 2005 by Wild as encompassing life-course environmental exposures (including lifestyle factors), from the prenatal period onwards [6–8]. The exposome can be structured following several logics, leading to defining various exposome domains (see Table 2). A common classification of the exposome distinguishes chemical, physical, psychosocial, (external) biological factors, as well as possibly behavioural factors (Fig. 1). Note that some authors include the biological responses to exposures (e.g., inflammation markers) in the exposome [10], and sometimes even endogenous factors, which we will not do here to distinguish the exposome from possible effect biomarkers that can also be influenced by genetic factors and diseases.

**Table 2:** The exposome domains

Logic and domains	Perimeter/definition/remarks	Examples
Physical–chemical-biological nature of the exposures*		
**Chemical factors**	Includes tens of thousands of factors, as well as mixtures. Many subcategories exist (man-made chemicals/POPs/PCBs…)	Benzene, bisphenol A, PCB153, drugs, diet…
**Physical factors**		Noise, heat/temperature, radiations, meteorological conditions, aesthetic factors
**Biological factors (extraneous)**	Note the specific case of mycotoxins (chemicals generated by biological agents)	Virus, fungi, prokaryotes (bacteria and archaea), contact with other large species…
**Behavioural/lifestyle factors**	This domain relies on the somewhat arbitrary distinction between “individual” factors and those related to the remote environment, thus ignoring the influence of the social and natural environments on human behaviours	Physical activity, diet-related factors (including alcohol consumption; these can also be seen as factors of chemical and physical nature), tobacco smoke (can also be seen as chemical factors), other addictions (illegal drugs, games, gambling), hygienic behaviours, sleep…
**Psychosocial factors**		Social capital, socioeconomic factors, education, stress
**Systemic factors**	Can be used to encompass a set of factors (possibly of different natures) sharing a common origin	Climate change, biodiversity loss, antimicrobial resistance…
***Assessment approach-oriented***		
**Biomarker-based exposures**	Mostly used for chemicals. Numerous matrices (blood, urine, hair…). Approaches can be targeted or not and may identify metabolites of the parent compound	Biomarkers of exposure to phenols, phthalates; cotinine as a biomarker of tobacco smoke exposure
**Dosimeter-based**	Often corresponds to physical factors	Temperature, noise, ultraviolet or γ radiation, personal exposure to fine particulate matter exposure…
**Environmental measures and models**	Typically weather-related parameters and atmospheric pollutants. Usually combine measurements or data on sources/emissions with deterministic or statistical modelling	Dispersion models or land-use regression models used for air pollutants
**Job-exposure matrix (JEM)**	Typically used in occupational settings. An approach combining environmental measures or data and data on job history	Occupational exposure to organic compounds or pesticides
**Questionnaire-based**	Typically used for dietary factors and tobacco smoke exposure. Sometimes used in conjunction with other approaches	Food frequency questionnaires, occupational exposures…
***Source/milieu-oriented***		
**Air pollutants**	Indoor and outdoor air can be distinguished	
**Water pollutants**	Includes many of the “chemicals” and possibly biological agents	Chlorination by-products (chloroform…), nitrates, drug residuals, specific pesticides…
**Dietary factors**	Includes the diet, diet contaminants and additives…	
**Soil contaminants**		Metals, biological agents…
**Occupational exposures**	Potentially includes the whole exposome	
***Internal/external*** [9]		
**Internal domain**	Processes internal to the body	Metabolism, endogenous hormones, body morphology, microflora, inflammation…
**Specific external**	Specific external exposures [includes physical, chemical, biological exposures, but not psychosocial factors]	Radiation, infectious agents, chemicals, diet…
**General external**	Social, economic and psychological influences on the individual	Social capital, education, psychological and mental stress, climate…
***Classification by Vermeulen *et al**. [10]		
**Physical–chemical**		Pesticides, persistent organic pollutants…
**Lifestyle**		Physical activity, drug use, diet…
**Ecosystems**		Greenspaces, food and alcohol outlets…
**Social**		Household characteristics, education…

^*^See Table 3 for more details on this grouping of exposome domains

The first applied publications referring to the exposome concept were published around 2010 [15]. However, if one accepts that studies in humans considering only a part of the exposome qualify as “exposome studies”, there have actually been “exposome-type” studies for decades. For example, at least since the 1980 s, air pollution studies simultaneously considered several air pollutants, such as particulate matter, sulphur or nitrogen dioxides, ozone…[16]. Even earlier, there were multi-exposure studies considering exposures from two families or more at least since the 1960s. In particular, in the area of cardiovascular health and of health consequences of dietary factors, studies simultaneously considered smoking, the levels of various lipids such as cholesterol, blood pressure or being overweight [17], or diet. Interest for the study of mixtures in humans exists at least since the 1990 s [18]. It remains that, until the early 2000 s, etiological studies in humans were almost exclusively single exposure studies. The studies of smoking effects on lung cancer by Doll and Hill initiated in the 1950s [19] are early examples of such studies – this example illustrates that epidemiologists in fact considered mixtures of exposures very early on in the history of epidemiology, but handled them as a whole (e.g., cigarette smoking or proximity to road traffic, as proxies of exposure to the thousands of chemicals composing cigarette smoke or the motors’ exhaust). Several drivers of a given health parameter are actually generally studied in the same population (e.g., Framingham [20] or ALSPAC [21] cohorts), but the corresponding results are most often reported in distinct successive single exposure publications. When it comes to the description of environmental exposures, human biomonitoring surveys considering several families of chemicals exist at least since the 1990 s [22].

The last twenty years witnessed the publication of many reviews on the exposome concept. These mostly covered issues related to exposure assessment, such as the relevance of mass spectrometry for exposure assessment [10, 23–25], the need or possibility for exposome research to consider the mechanisms underlying effects of exposure and to rely on omics tools [26–31], or the connection with toxicology and the adverse outcome pathway concept [30, 32, 33] (see Supplementary Table 1). Very few of these reviews delved into the details of study design beyond issues related to exposure assessment nor attempted to connect exposome research with other multi-exposure approaches, designs and issues such as human biomonitoring [34], health impact/risk assessment and studies on the modification of environmental exposures (e.g., through interventional research). Thus, so far, the exposome promises have mostly been formulated in terms of the ability of exposome research to unravel the causes of diseases, and in particular chronic diseases [9], as well as the underlying mechanisms [8, 10, 29]. We aim here to fill this gap and propose to frame exposome research as centred around four key aims highlighting the relevance of exposome research for public health.

**Fig. 2:**
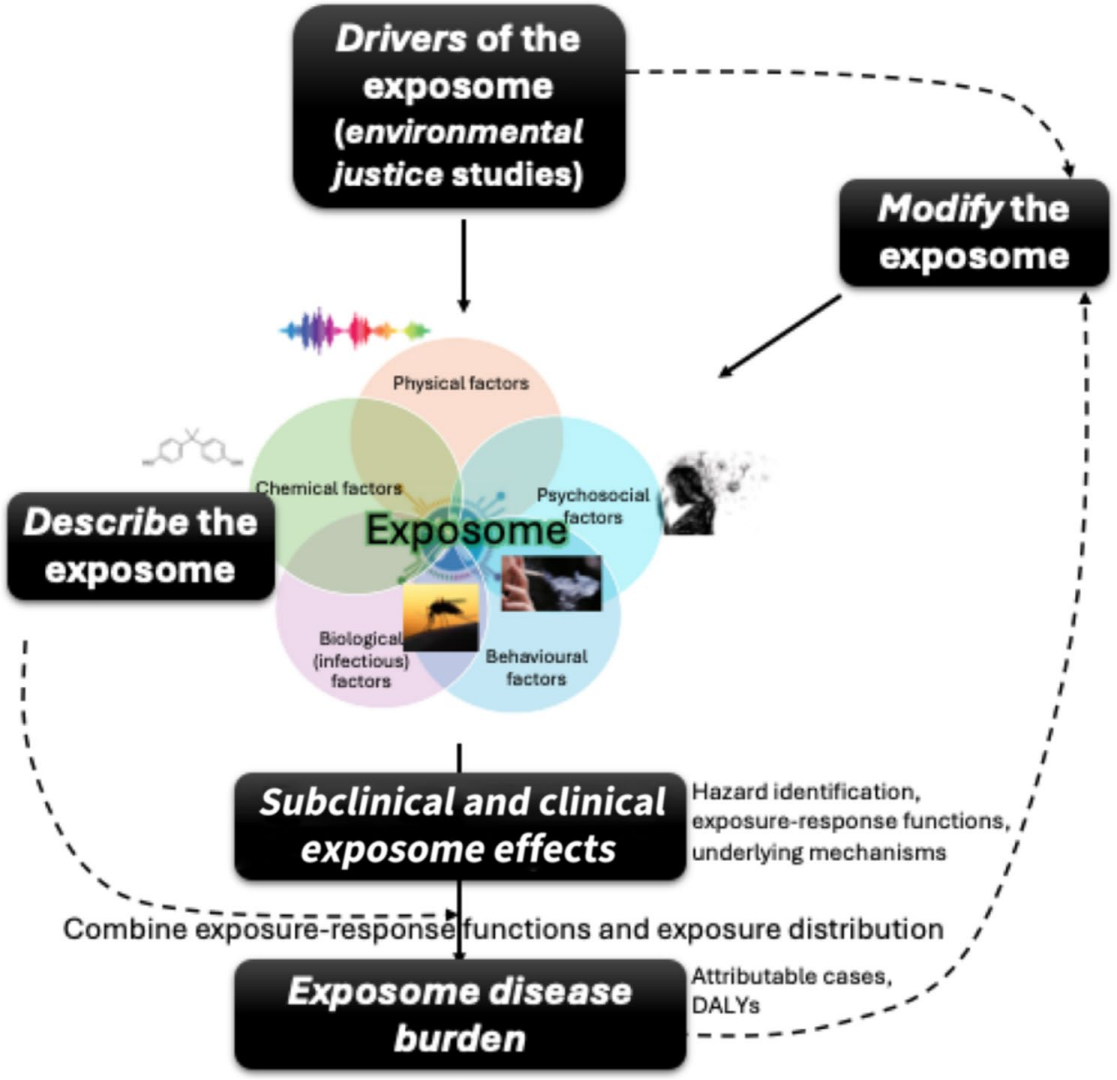
Key aims of exposome research: describing the exposome, its drivers, its subclinical and clinical consequences, the corresponding population burden, and identify ways to modify the most noxious exposures

These aims that are or could be tackled by exposome studies include first a descriptive aim, consisting in describing exposures and assessing correlations between exposures and within-subject (temporal) variability in exposures. This include “environmental justice” studies describing associations of the exposome with sociodemographic and geographical factors and the drivers of exposures. A second aim is to enhance our understanding of the possible biological (subclinical and clinical) effects of environmental exposures (etiologic aim, or hazard assessment in the risk assessment terminology), typically providing exposure–response functions, often in the form of relative risks. A third and more public health-oriented aim relates to the quantification of the health impact of the exposome and the establishment of a ranking of exposures in terms of their population health impacts (health impact assessment, environmental burden of disease aim or equivalently, risk assessment, typically providing estimates of disease cases and premature deaths attributable to exposures). Finally, if effects or impacts of components of the exposome are convincingly demonstrated, a further aim is to establish science-based levers and guidelines on how to modify these components of the exposome to improve human health (interventional aim; see Fig. 2).

Here, we aim to provide a structuration of exposome research around these four promises of exposome studies, on the related challenges and obstacles as well as on the potential for exposome studies to overcome some of the limitations of single exposure articles. We will successively discuss the above-mentioned aims, drawing lessons for the design of future exposome studies. We will illustrate our points relying on past exposome projects and simulation studies.

## Descriptive aim: assessing and describing the exposome

### Describing the human exposome: aims and challenges

A description of the exposome should provide information on: the levels of all exposures; the spatial and temporal variations of these exposures at the individual and population levels; the assessment of the correlation between exposures and, more broadly, the co-occurrence of exposures through the identification of exposome patterns; the understanding of the drivers of these exposures, including the sociodemographic, geographic or behavioural drivers. Many studies are limited to this descriptive aim, without centrally considering etiologic aims. They consider chemicals assessed from biological matrices (“biomonitoring studies”, such as NHANES [35] and similar studies in some EU countries and elsewhere), or in water or food samples [36], atmospheric pollutants and climate-related factors [37, 38] (emission registries of atmospheric contaminants, large-scale environmental measurements and modelling such as global circulation models) and so-called lifestyle and behavioural factors (e.g., dietary behaviours [39], studies on smoking prevalence [40]…).

Challenges of descriptive exposome studies include the fact that they tend to be conducted separately in each exposome domain, hindering the assessment of correlations across these broad exposure categories, such as between chemicals and weather-related factors. Exceptions exist, such as NHANES study, providing information on dietary behaviours, smoking and biomarker-based body contamination by chemicals [35]. Other challenges include those related to population representativeness and selection bias, and to within-subject variations in exposures.

### Descriptive exposome studies: design-related considerations

*Population sampling:* When the aim is to compare the exposome across locations, using harmonized methods is crucial. This applies both to exposure assessment tools (exposure matrix, analytical approach) and to population sampling (and/or site sampling if environmental measurements are performed). The choice of the population sampling method depends on the desired generalizability of the results and on the specific comparisons planned. Random population sampling from a well-defined sampling base is required when the primary aim is to describe the exposome. Oversampling specific subgroups with an expected low size or participation rate may be relevant. Multistage random sampling schemes clustered on other factors such as urban or rural status can also in principle be used. Using such multistage sampling implies to correct for the complex design in the descriptive and possibly etiologic analyses [41]. This corresponds to the design of NHANES study in the USA [35] and is also commonly used outside the exposome context to accurately describe e.g., disadvantaged social groups [42, 43]. A few hundred to a thousand subjects may be enough for such descriptive studies. This number should roughly be multiplied by the number of specific groups (areas, socio-economic categories…) if a fine description of the exposome in target groups or if differences in exposome across groups are to be tested, and possibly even more if corrections for multiple testing are planned.

*Assessment of the exposome:* Challenges in the assessment of the exposome include the large number of exposures to consider and the multiplicity of their sources and exposure pathways and routes; the within-subject (temporal) variability of many exposures (discussed below); the lack of a unique assessment matrix; and the fact that assessments sometimes rely on proxies of exposure rather than the actual exposure of interest (e.g., urinary metabolites of benzene instead of benzene exposure; we use here the strict definition of exposure as the amount of an environmental factor that is in contact with the boundaries of the body).

Starting with this last point, the definition of what exposure means is in favour of relying on environmental rather than human sampling to describe the exposome. That is, analysing drinking water for the presence of pesticides or chlorination by-products can, if the information is combined with questionnaire data on drinking behaviours and other behaviours implying a contact with water, provide an accurate estimation of exposure to these chemicals from drinking water. However, the multiplicity of sources and exposure routes of pesticides implies that this approach would need to be repeated for diet and air contamination. Sampling water, air and food in a large number of subjects and collecting detailed enough information on the related behaviours to provide an estimate of exposure considering all exposure pathways can be very cumbersome. Conducting a biomonitoring survey on the same number of subjects implies to only rely on a single matrix (urine, if the pesticides or their metabolites can efficiently be detected in urine) and does not necessarily require to collect detailed information on water use and consumption, diet, time spent indoor and outdoor in various locations… Probably for this reason, many descriptive exposome studies rely on human biomonitoring (although environmental monitoring also exists, generally not in all milieux in parallel, limiting the ability to combine the information from each milieu). However, urinary levels correspond to excreted concentrations and not exposure, and the connection between exposure and excreted levels depends on complex toxicokinetic mechanisms and individual characteristics (body mass index, genetic polymorphisms in xenobiotic metabolizing genes…) and behaviours (physical activity, water consumption…). Pharmacokinetic modelling can be used to obtain an estimate closer to exposure from urinary concentrations, or from blood levels (see also below).

Some early biomarker-based descriptive exposome studies had to assess different exposures in different subjects because large volumes of urine or blood were required to assay a small number of chemicals. However, with the decreased volume of biospecimens required for each chemical analysis, it is now possible to try to assess a very large number of exposures in the same individuals, as reviewed elsewhere [24, 25]. This allows evaluation of between-exposure correlations and is less cumbersome because the total number of subjects recruited is smaller for a similar number of biological assays (i.e., it is cheaper to assay 300 exposures in 1000 subjects than to recruit two groups of 1000 subjects and assay 150 exposures in each of them). The cost may be a decreased participation rate because of a possibly increased participation burden.

There is not a single biological matrix (e.g., circulating blood or hair) or approach (e.g., environmental models) that can be used to characterize the exposome. Thus, studies relying on a single matrix or exposure assessment tool generally only capture a fraction of the exposome, and studies not restricted to a specific exposome domain (e.g., chemicals that can be assessed in biological matrices) need to combine several types of exposure assessment tools. For example, the HELIX project assessed chemicals or their metabolites from biomarkers in urine and blood; factors of the “urban exposome” (particulate matter and other atmospheric pollutants, temperature, greenspace exposure) from environmental models and measurements, as well as in personal dosimeters for population subgroups; and socioeconomic factors (such as educational level) and lifestyle and behavioural factors (diet, physical activity, sleep, exposure to tobacco smoke) from questionnaires [44, 45]. This diversity in assessment approaches can increase the burden on study participants, thereby limiting participation, inducing selection bias and increasing the rate of missing data. Thus, there is a trade-off between the extent of the exposome characterized and the representativeness of the population. In principle, for descriptive studies, representativeness should be favoured, while a different choice could be made for etiologic studies. In this logic, a “partial” participation should be favoured over a lack of participation (which may increase selection bias). If the correlation structure of the exposome is not of core interest, then the exposome could be divided into various components whose assessment would be (ideally, randomly) assigned to subjects not willing or able to fully participate, with a core set of sociodemographic, anthropometric and health data being collected in all subjects to study and possibly correct for selection bias [42, 43].

### Within-exposure vision: temporal variability of the exposome.

**Fig. 3:**
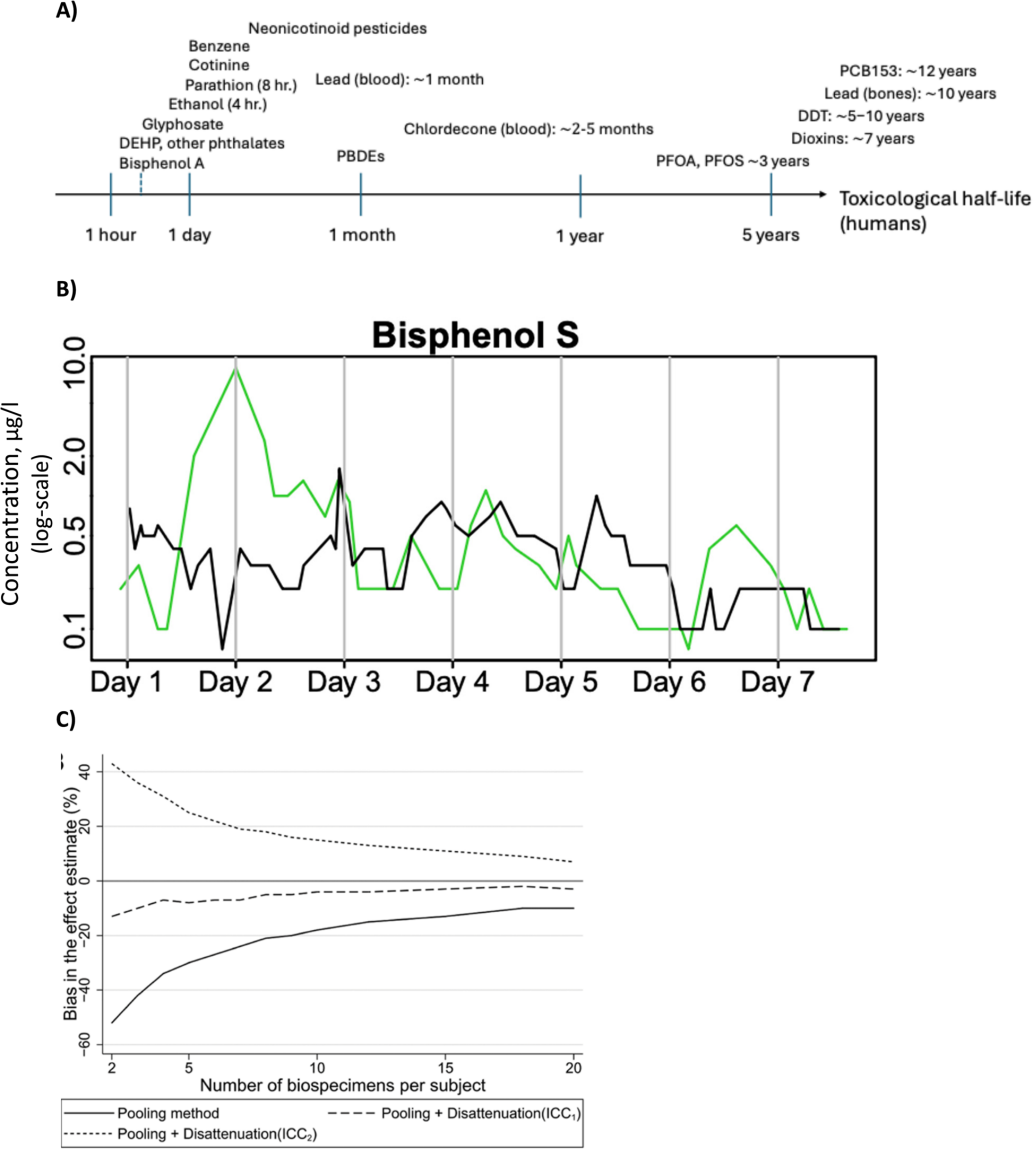
Issues related to the temporal variability in exposures. **A** Approximate half-life of select chemicals in humans. **B** Variations in urinary Bisphenol S (a chemical contaminant) concentrations in two pregnant women in whom a sample of all micturitions were collected during a week (each curve corresponds to a woman; note that concentrations vary by a ratio of about 1 to 20 in one woman and 1 to 100 in the other woman between samples collected in the same week). **C** Bias in the estimated effect of an exposure influencing a health outcome according to the number of biospecimens collected in each subject to assess exposure and to the analytical approach [47] in the case of a compound with an intra-class coefficient of correlation of 0.2

A large fraction of the exposome strongly varies temporally within a subject (see Fig. 3A for examples of half-lives of chemicals). This is the case of psychosocial factors such as stress, of many physical factors such as temperature, particulate matter and other atmospheric pollutants, of exposure to some electromagnetic fields, of dietary exposures and tobacco smoke exposure as well as of most of the chemical factors currently marketed: bisphenols and phthalates for example typically have half-lives in the body of a few hours. Even compounds with relatively long half-lives, which are sometimes stored and released by fat tissues, have circulating and possibly organ-specific levels that can strongly vary within a subject, at least over periods of a few years, for example because of a delivery, of women breastfeeding their child or of a change in weight [46].

Characterising the within-subject temporal variability in the exposome is important for descriptive purposes, because the exposure distribution will be biased for exposures with strong within-subject variability if only a spot (short-time) assessment of exposure is performed in each subject (the average level may be correctly estimated, but between-subject variability is expected to be biased). It is also essential for etiologic exposome studies. Describing this variability, as well as other important phenomena related to the distribution of the compounds of interest and their metabolites in the body, their metabolism and excretion, is the task of toxicokinetics. However, toxicokinetic parameters may vary between humans and the animal models used in toxicology, as is the case e.g. for polyfluoroalkyl substances (PFAS)[48], so that performing toxicokinetic studies in humans (fulfilling all ethical requirements) or at least describing the variability of exposure proxies in humans can be useful. This variability implies to be able to estimate exposure proxies over relatively long periods in each subject, through repeated biospecimens collection or dosimeters use. If describing the within-subject variability is not the main aim, then repeatedly assessing exposures only in a random subgroup of the study population is an option to decrease costs. In Helix project, for example, subgroups of about 150 pregnant women and children, representing 10–15% of the study population, followed an additional protocol aiming at characterizing the variability of the exposome. These pregnant women provided 3 urine samples per day during two pregnancy weeks [49] and repeatedly carried dosimeters [50], which among other things allowed to characterize the within-subject variability of exposures in this population and to estimate the number of biospecimens allowing to limit exposure misclassification [49, 50].

### Between-exposure vision: exposome structure

**Fig. 4:**
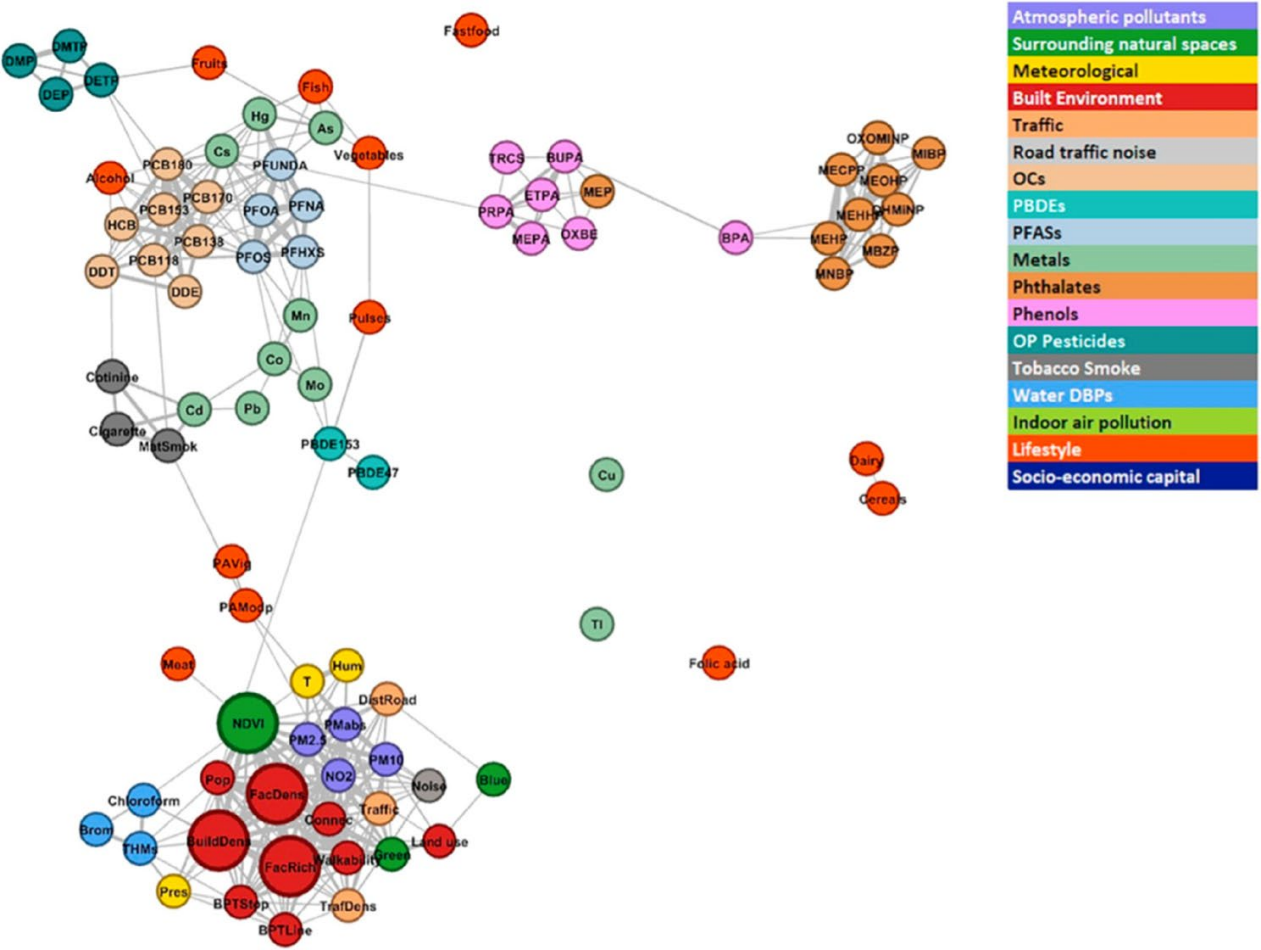
Correlation structure of the (pregnancy) exposome, as assessed in 1301 pregnant women from Helix cohorts. The size of the nodes is proportional to the number of correlations were > 0.5 outside the exposure group and the length of the edges is proportional to the inverse of the correlation (the higher the correlation, the shorter the edge length) between exposures. The colour of the nodes represents the pre-defined exposure groups. All connections between exposures represent correlation levels of 0.10 or above. From Tamayo-Uria et al. [51]

The exposome can have a high to very high dimension, beyond several thousand or tens of thousands of exposure variables (Table 3), although most published studies do not go beyond a couple hundred exposures. Correlations between exposures tend overall to be low when a large number of exposure families are assessed [51]. Correlations are expected to be higher *within* exposure families (defined by their chemical nature or source) than *between* exposure families. In HELIX cohorts, which covered several EU countries, a few pairs of exposures had correlations greater than 0.9; a few exposure families had median within-family correlations around 0.5 (e.g. for atmospheric pollutants and for per- and polyfluoroalkyl substances (PFAS)) and possibly higher within some areas, while correlations were much lower within the other families (e.g., 0.14 among phenols; see Fig. 4) [51]. The median correlations *between* exposure families (e.g., between a metal and a PCB congener) were much smaller, rarely exceeding 0.3 [51]. Describing such correlation structures is relevant, and can e.g., allow making hypotheses regarding the sources of exposures or behaviours associated with them. It is also relevant for etiological studies (see below). Correlations between exposures reduce the underlying dimensionality of the exposome. In Helix, 95% of the variability in 209 exposures (taken at two time points) could be explained by 65 principal components [51].

**Table 3:** Estimation of the number of factors in each exposome domain

**Exposome domain**	Inventory of factors, estimated number and references
Chemical factors	350,000 for marketed chemicals [11] Non-marketed chemicals (include components of diet of natural origin)
Physical factors	Meteorological factors (temperature, humidity, wind-related conditions, flooding,), ionising radiations (α, β, γ, neutron: 4), non-ionising radiations (UV, visible light, infrared, micro- and radio-waves, extremely low frequency radiation: 6), noise, vibrations, aesthetics, exposure to greenspace and blue space, food environment… Total, approximately 20
Biological factors (extraneous)	Prokaryotes (bacteria and archaea, 10^6^) [12], unicellular eukaryotes (10^6^; see [13]), virus (possibly a total of 10^31^, including about 18,000 species documented in the ICTV in 2026*, out of which 451 are considered “human” viruses [14]), contact with other large species including wildlife and domestic animals…
Behavioural factors	Physical activity, diet-related factors (including alcohol consumption; these can also be seen as factors of chemical or physical nature), tobacco smoke (can also be seen as chemical factors), other addictions (illegal drugs, games, gambling), hygienic behaviours, sleep…
Psychosocial factors	Social capital, income-related factors, education, non-chemical and non-physical occupational factors, education, stress, origin
Systemic factors	Climate change, biodiversity loss, antimicrobial resistance…

^*^ICTV International Committee on Taxonomy of Viruses; see https://ictv.global/taxonomy

When examining correlations, one needs to consider that correlations may be driven by the methods used. Indeed, exposures measured with similar instruments or in the same biological matrix may exhibit “inflated” correlations, and symmetrically, exposures with different timings of measurements or analysed by different laboratories can exhibit correlations biased towards the null.

Descriptive analyses of the exposome can be conducted, for example, using correlation plots, principal component analysis, network analysis, or cluster analysis. Cluster analysis has the capacity to identify several groups of individuals, each one characterized by a specific exposome profile [52].

### Social contrasts in exposures: exposome studies informing environmental justice

The description of the structure of the exposome is a first relevant step before considering its associations with other individual or community-level characteristics. Associations can be sought with factors causally “upstream” of human exposures, such as the sources of exposures, locations, behaviours, sociodemographic or biological characteristics possibly influencing the exposome (to understand its possible drivers and possibly identify ways to modify it). They can also be sought “downstream” of the exposome, to understand its consequences on subclinical and clinical factors and on society (see below and Fig. 2). Examples of studies connecting the exposome with “upstream” factors include studies on the relation between cosmetic use and exposures to compounds suspected to be present in cosmetics, such as parabens or phthalates [53, 54], studies on dietary patterns and exposure to food additives and contaminants [55], and studies on the distribution of the exposome across sociodemographic characteristics. The latter studies are connected to the broader topic of environmental justice. Environmental justice emerged as a social and political movement and led to scholar studies interested in describing whether environmental exposures vary according to ethnicity or social classes [56]. In addition, these studies can assess the extent to which these social inequalities contribute to disparities in disease burden across these groups, as currently done e.g. for single exposures such as smoking [57]. Such studies are also informative when designing interventions to limit the health burden incurred by the exposome (see below). Many early environmental justice studies started as single exposure or single exposure family studies (typical examples include studies of social inequalities in smoking, vicinity to landfill sites [58] or air pollution exposure [59]). More recent biomonitoring studies or exposome cohorts have started to document the heterogeneity in exposure to multiple exposures across educational levels [60] or in relation with income [61].

The first exposome studies on environmental justice issues did not correct for multiple testing, but this could be considered. Alternatively, statistical clustering techniques could be used [62]; it is expected that exposures sharing similar sources or exposure routes or that co-occur spatially for other reasons tend to cluster. Like for purely descriptive studies, a sample size between a few hundred and a few thousand individuals may be enough for such studies, depending on the distribution of the socio-economic factors of interest and on whether subgroup analyses are planned; complex sampling designs (e.g., multilevel random sampling), allowing to preserve representativeness of final results while optimizing sample size, may be relevant.

We will now move to questions related to the identification of the downstream consequences of the exposome that is, its subclinical and clinical effects.

## Etiologic aim: Relating the exposome to biological parameters (hazard identification)

### Challenges of etiologic exposome studies

A core aim of exposome research deals with the identification of biological (subclinical and clinical) effects of environmental exposures, which is also termed toxicodynamics. This etiologic aim includes the identification of the biological mechanisms of action of exposures on health. An important long-term output of such studies, together with single exposure etiologic studies in humans and in vivo and in vitro approaches, consists in the lists of factors that probably or certainly influence health or act following specific mechanisms, together with the corresponding exposure–response functions. From a risk management perspective, such studies feed the process of hazard identification, consisting in defining which exposure factor belongs to which hazard categories, as defined e.g. by the GHS, or Globally Harmonized System of classification and labelling of chemicals of the United Nations or the EU CLP regulation. Examples include the list of carcinogens established by IARC, the International Agency for Research on Cancer, or that of substances of very high concern developed in the context of EU REACH regulation [63], or in scientific contexts [64] (see Fig. 10 for another example).

Generally, etiologic exposome studies in humans have to face at least five challenges: the curse of dimensionality (or sparsity), the challenge of correlation between exposures, a trade-off between breadth and depth, the challenge of measurement error and challenges related to pharmacokinetic bias. As we discuss in the last section, only the curse of dimensionality and the trade-off between breadth and depth are present in exposome and not in single exposure studies.

**Fig. 5:**
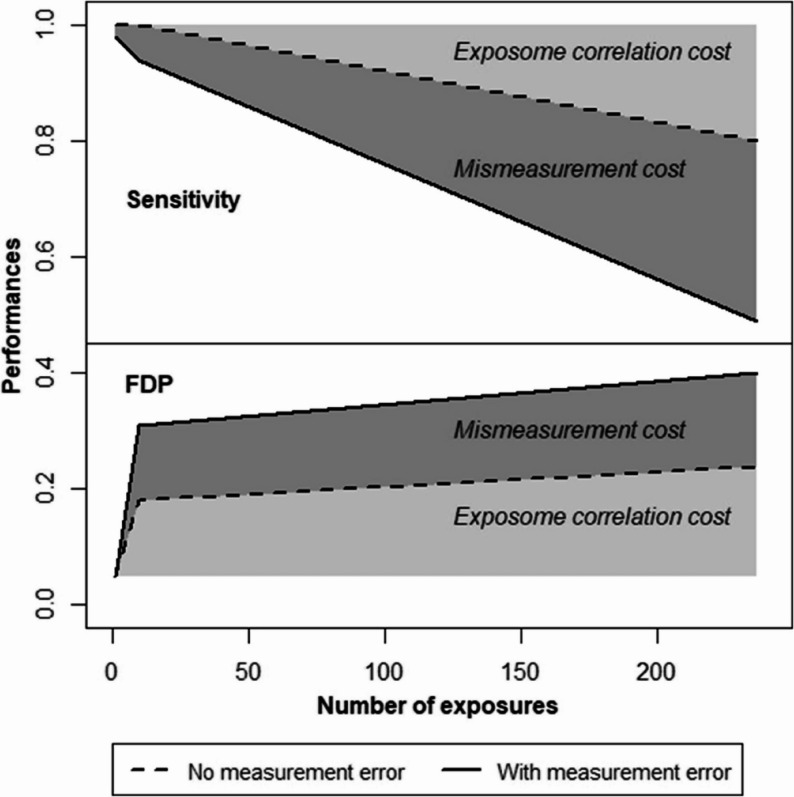
Decreases in sensitivity and increase in false detection probability (FDP) as the number of exposures considered increases, in the absence (dotted lines) or presence (continuous lines) of measurement error in exposures. Simulation study from Agier et al. [67]

The challenge (or curse) of dimensionality [65, 66] is related to the fact that, as the number of considered exposures (or, in mathematical terms, the dimension of the considered space) grows, if the number of observations (the number of individuals n) remains constant, then the data become more sparse. The consequence is that estimation of the disease probability associated with a given combination of exposure levels has to rely on a very small number of observations, leading to possibly inaccurate estimations, and that the identification of a true (causal) predictor of disease among many other correlated exposure variables is made more difficult as the number of exposures increases without the number of observations simultaneously increasing. This is a well-known issue in statistics [66]. It relates to the fact that, to inform the management of environmental hazards, exposome studies need to identify causal predictors of health, not simply build models predictive of disease risk relying on non-causal variables. Agier et al., in the specific context of exposome studies, highlighted that at fixed sample size, the sensitivity to detect exposures causally linked to the outcome decreases, and the false detection rate increases, as the number of exposures increases [67] (see also Fig. 5). It can be shown that this effect is a consequence of the increase in the dimension in the context of (even moderate) correlations between exposures [2]. The situation is worsened when there is measurement error in the exposures, due to within-subject variability in exposures when only a spot exposure measurement is conducted. This within-subject exposure variability is due to the short biological half-life of many chemicals and to the strong variability in behaviours and environmental factors driving many exposures. It is expected to induce a bias towards the null, at least in the context of so-called classical-type error [68, 69]. The two sources of bias (the curse of dimensionality and measurement error in exposure) add up. As an illustration, in a simulation study of 1200 subjects, the sensitivity to identify true predictors (i.e., the proportion of causal variables actually identified) decreased from 100% when 10 exposures (including one true predictor) were considered, to 80% when the number of exposures increased to 237, and to 49% if these 237 exposures were assessed with some (realistic) amount of measurement error. The false detection proportion (proportion of selected variables that are not causally linked to the outcome) increased from 18 to 30% and 40%, respectively [67]. Thus, with this sample size of 1200 individuals and about 240 exposures modestly correlated, only half of the true predictors were identified, and the list of hits included 40% of false positives: the combined impacts of the curse of dimension, of correlation between exposures and of exposure misclassification is expected to lead to underpowered, biased and overall inefficient studies (Fig. 5).

The trade-off between breadth and depth corresponds to the fact that, as the number of exposures considered in a single publication (the study *breadth*, or coverage in terms of exposures) increases, it becomes more difficult to tailor study design and analysis to each single exposure, and to investigate exposure-specific bias or biological mechanisms (the *depth* of the study). For example, an exposome study may rely on a single set of adjustment factors used for all exposures (while the list of potential confounders may be exposure-specific [70]); it may test a single simple functional form for all exposure response functions, such as an affine function, to limit dimensionality increases, disregarding codings of exposures that could allow for non-monotonous exposure-outcome associations.

### Design of etiologic exposome studies

We will focus on exposome studies considering a single health outcome or biological parameter at a time (e.g., the molecular initiating event of an adverse outcome pathway, see [32]), leaving aside the case of multiple health outcomes [71] or biological parameters, as would be the case if e.g., omic data were handled in an agnostic way.

*Study design: *Cohort studies appear to be the best-suited design to investigate the health effects of the exposome. This design parallels that of toxicological animal studies, with the key difference that exposure is controlled in toxicology, while it is observed in most cohort studies in humans. Indeed, a prospective follow-up of participants, with repeated measures of exposures and health parameters, can capture life-course influences. In contrast, due to the temporal variability in many exposures, and to the potential for diseases to modify behaviours related to exposure, retrospective study designs are likely to entail large measurement error in exposures (they are not likely to accurately reflect exposures in the toxicologically-relevant time window, some time before disease occurrence) and might even suffer from reverse causality. Of course, for some exposures such as regulated air, food or drinking water pollutants, a retrospective assessment can be considered because of the existence of monitoring networks or large-scale monitoring studies. However, such strongly monitored exposures currently correspond to a small fraction of the exposome in most countries, and generally to exposures already identified as being harmful for health; moreover, a proper assessment of these exposures would often imply to combine these measurements in air, water or food with individual information on behaviours influencing exposures, such as drinking water or showering habits for drinking water pollutants, which are more difficult to assess retrospectively. In the “general”, non-exposome setting (typically for genetic risk factors), case–control studies constitute an appealing design in the case of rare diseases such as most cancers; however, the above-mentioned issues related to the quality of exposure assessment call for sticking to a prospective design – i.e., to very large exposome cohorts (see below). Applying a retrospective design in an exposome setting will lead to very strong differential sensitivity across exposures. In other words, with such a design, the effects of causal exposures that can efficiently be assessed retrospectively, such as maybe active smoking or exposure to regulated atmospheric pollutants or to persistent compounds, may be more likely to be highlighted than those of causal exposures for which retrospective assessment entails strong (attenuation) bias. The latter factors include dietary factors and non-persistent chemicals, which correspond to the majority of currently marketed chemicals. Similarly, the case-crossover design, which is in principle relevant to investigate transient effects of exposures, is a retrospective design, which limits its ability to efficiently assess a large number of exposures.

These transient effects are best investigated with prospective designs, such as a panel study, which is nothing else than a cohort design with a short follow-up duration. The BAPE study implemented this design, collecting personal exposure to dozens of air pollutants, as well as biomarkers of exposure and of effect, in five occasions in the same subjects [72, 73]. These studies can incorporate an intervention arm aiming to modify exposures, possibly with randomization. Such interventions are likely to only target one component of the exposome, such as airborne, waterborne or dietary exposures, whose effects are investigated, generally on subclinical parameters rather than clinical outcomes. For example, in an intervention among 50 men, volunteers were asked to abstain from going in a swimming pool for a week, after which they were asked to swim in a chlorinated swimming pool for 40 min. Exposures to chlorination by products (trihalomethanes) and levels of biomarkers of effects were assessed before and after swimming [74].

The exposome definition, referring to an exposure window starting at conception, as well as DOHaD (developmental origins of health and disease) concept [75], make pregnancy and pre-pregnancy cohorts particularly relevant to investigate the health effects of the exposome. Animal studies documented effects of exposures occurring before fertilization [76]; this is the case of a few observational studies in humans [77]. For these reasons, it would be worth considering not to limit the assessment of the exposome to the period starting at conception, but to try to consider the effect of the parental exposome on the health of children and grand-children. Historically, birth cohorts (with recruitment at birth) were first conducted, not allowing to efficiently characterize the pregnancy exposome; from the late 20th Century at least, pregnancy cohorts (with recruitment typically in the second or third trimesters of pregnancy) were set up. Examples of – much rarer – cohorts with peri-or pre-conceptional recruitment, and often rather short-term follow-up so far also exist [78–81]. Using them to consider health outcomes in young adults or later takes patience – prospective cohorts from birth to old age require a lifetime to be built. To limit long-term attrition, passive follow-up of disease incidence through nationwide healthcare databases, which are increasingly available, is an option. The prospective long-term design does not preclude consideration of emerging exposome components, at least for the chemical and biological components of the exposome. Indeed, collection at inclusion and long-term storage of biological and environmental samples (e.g., dust samples collected in the homes of participants) allows to investigate new environmental factors as new hypotheses emerge, even after the end of population follow-up. This approach provides accurate information on exposures as they were before the time of disease occurrence, just like in a case-cohort or nested case–control design [82].

“Accelerated longitudinal designs”, which combine different cohorts starting at different ages, is an alternative to a single large exposome cohort [83]. They are clearly not equivalent to the information provided by a single long cohort, as the accelerated longitudinal design only allows assessing effects of exposures that unfold during the follow-up of each cohort, and not effects across the various cohorts considered.

More specific design considerations include deciding the study setting and population as well as exposome assessment tools.

*Study areas and population*: Deciding the study setting is critical. The main concern here should be avoiding selection bias. Contrarily to descriptive exposome studies (see above), recruiting a representative population may not be essential, as generalizability of results is not necessarily achieved through representativeness [84]. Recruiting a population sample representative of the general population is also not always the most statistically powerful design. As an illustration, focusing on a population of a specific age group (therefore not representative of the whole population) may be more efficient than covering all age groups with the same total sample size. This is because, in a context where different effects can be expected at different ages, the age-specific sample size, and hence the statistical power, is lower if all age groups are covered. Similarly, like in other etiologic studies, excluding specific subgroups (e.g., active smokers or overweight subjects), may be relevant to limit confounding (or exposure misclassification of exposure to air pollutants, in the case of the exclusion of smokers, if pollutants are assessed from personal dosimeters) by these factors. This would be done at the cost of not allowing to study effect measure modifications with the factors defining these subgroups, which would anyway probably require very large subgroups in an exposome context.

Regarding study areas, if there is a single recruitment area of rather small size (e.g., an urban area), exposure contrasts to some components of the exposome, such as outdoor air pollutants, may be small, limiting statistical power. Considering multiple centres/areas is an option that can allow expanding exposure ranges (and possibly increasing the number of subjects recruited in a short duration, depending on the target population). However, multicentre studies introduce analytical challenges in etiologic studies. Indeed, centre is usually a strong determinant of the exposome or of some of its components (weather-related factors, air or water pollutants, noise…), so that associations based on the between-centre variations have the risk of being confounded by unobserved variables that are related to centre. Thus, in many cases, centres are analysed separately and the results are combined with meta-analyses. Replication of an association across centres with possibly different confounding structures can make observed associations more convincing [85, 86], but this requires having enough statistical power within each centre. Alternatively, analysts use models with centre considered as fixed effects (i.e., through classical adjustment), which in practice excludes between-centre differences and implies that effect estimates rely only on within-centre associations [87]. Adjusting for centre using random effects allows for using both within-centre and between-centre contrasts in exposure. However, it may still be advisable to partition associations into *between-centre* and *within-centre* components to better interpret the results [88]. In any case, designers of etiological exposome studies should be cautious in considering a large number of study sites – also because of the logistic burden implied by a possibly long-term follow-up in many areas. An alternative consists in relying on a (possibly very) small number of large areas, such as whole regions or countries. This may, again, raise important logistic challenges in terms of population follow-up and exposure assessment.

*Exposure assessment—when:* When to measure exposures and how frequently are other key design questions. Assessing exposures with a strong within-subject variability (which correspond to most current exposures) at a single time point still corresponds to the most frequent approach. This is probably a convenience choice related to the perceived burden of repeated within-subject exposure sampling and to the lack of knowledge regarding its relevance. This reliance on a spot (short-term) assessment of exposure has been shown to induce a strong bias in exposure response functions, corresponding to a bias towards the null in the context of classical type error. The amplitude of the bias corresponds to 1 minus the intra-class coefficient of correlation (ICC) of the considered exposure/biomarker [69]. This corresponds to an expected attenuation bias of 80% (i.e., the observed association will in expectation correspond to one fifth of the true association) if the exposure has an ICC of 0.2 [68], a value observed for the assessment of bisphenol A in spot urine samples during pregnancy. Therefore, when multiple exposures are measured at a single time point, the sensitivity of the exposome study is much weaker for the exposures with the largest within-subject variations. In other words, disregarding any other source of bias, a study of a given sample size is much more likely to identify effects of a PCB (a well-known and now banned contaminant whose blood levels have a low variability) than of a bisphenol (a more recent family of contaminants whose internal levels have much higher variability). This differential (across exposures) exposure misclassification implies that “null” (non-significant) results need to be interpreted with even greater care than in the single-exposure context.

**Fig. 6:**
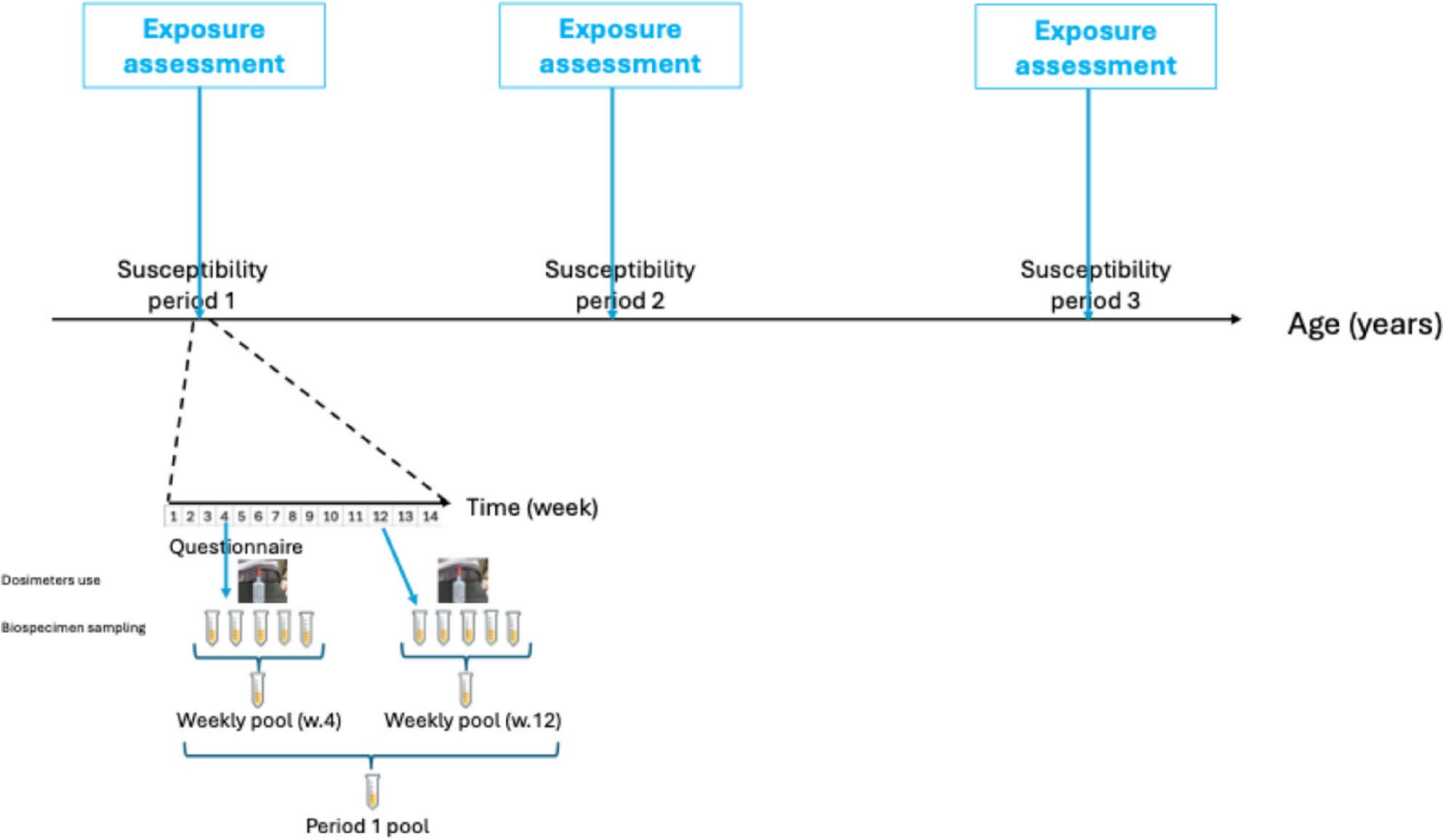
Illustration of a possible strategy of exposure assessment in an etiological exposome cohort. It is assumed that researchers targeted a priori 3 possible susceptibility periods; during each of them (which may last from a few weeks to a few years), a series of exposure assessment weeks are conducted in each subject, each consisting in questionnaires on behaviours and exposures (and possibly health), in repeated sampling of biospecimens and in the use of dosimeters (for exposures that cannot be assessed from biospecimens)

Fortunately, there is a solution to exposure misclassification due to within-subject variability. It consists in repeating the assessment of exposures across time for each subject [68, 69] (see also Fig. 6). For exposures that are assessed from biomarkers, this implies to collect several biospecimens per subject over time, and either assess the biomarker in each biospecimen or pool the biospecimens within subject before assessing the biomarker level. This latter approach (termed the “within-subject biospecimen pooling” [68]) allows to decrease bias without increasing assays costs, but does not allow to characterize the within-subject exposure variability. For exposures assessed from dosimeters (such as temperature, specific air pollutants), this implies to have the dosimeter being used several times for each subject, or to be carried during a long period of time, which in practice is currently difficult beyond a couple of weeks for many exposures. This approach has been shown to be theoretically efficient both in the single exposure [68] and in the exposome [67] contexts. It has been implemented, e.g., in SEPAGES exposome parents-child cohort, in which an average of 42 urine samples per woman have been collected during pregnancy [89], and in BISC cohort [90]. The collected data can then be analysed using measurement error models, such as regression calibration [47, 68], or by models that ignore the repeated exposure assessment if these exposures are averaged within subject. This exposure assessment approach consisting in repeated use of dosimeters and repeated biospecimens sampling can be repeated for each a priori selected window of sensitivity (Fig. 6). For exposures that are derived from external measurements or models, such as specific atmospheric pollutants, covering any a priori relevant exposure window can be achieved without additional effort, often at the cost of disregarding indoor air exposures, thus assuming that they are strongly correlated with outdoor levels, or, at least, that ignoring indoor levels does not bias estimates of associations. If personal measurements are preferred for air pollution, meteorological factors or noise, then the above-mentioned approach of relying on repeated exposure assessment is advised [69], unless only effects of exposures over a single short time period are of interest. Otherwise, the possible gain resulting from the increased spatial resolution provided by the personal measurement may be offset by the bias possibly induced by not targeting the toxicologically-relevant exposure window.

The issue of differential exposure misclassification implies that one should be cautious with approaches allowing to provide an estimate of a very large number of proxies of exposures, at the cost of a decreased accuracy in the assessment of the exposure. For example, untargeted metabolomics allow to strongly increase the number of exposures assessed, but sometimes providing a qualitative assessment of the exposure status (detected yes/no) rather than a quantitative assessment. Similarly, other omics layers may be used as indirect exposure proxies of specific exposures. Increasing strongly the number of exposures considered in an etiologic study while decreasing the accuracy of the assessment of these exposures and without strongly increasing sample size is expected to lead to the above-mentioned differential sensitivity across exposures and to a decrease, rather than an increase, in the sensitivity of the study (see sample size below).

**Fig. 7:**
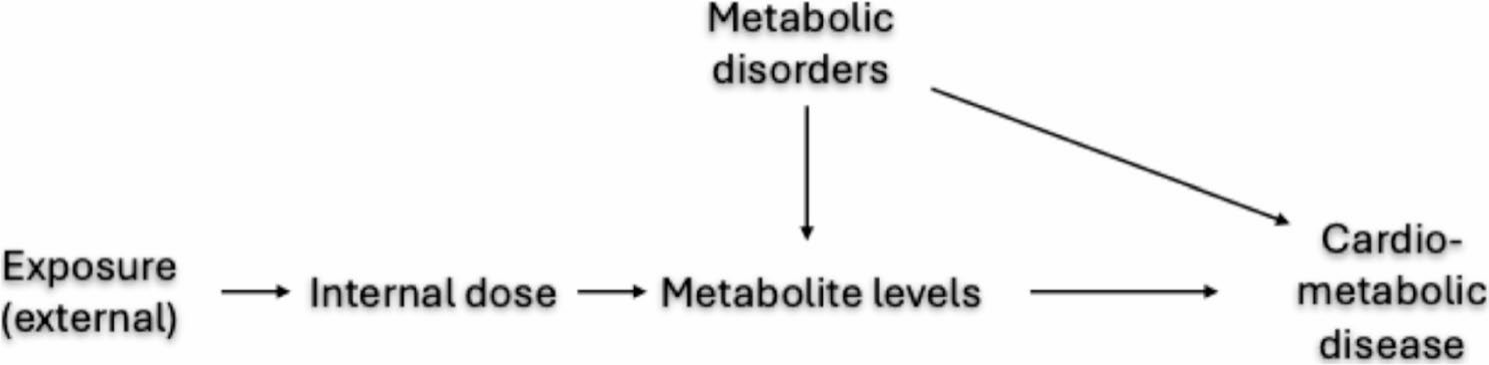
Illustration of the confounding bias that may arise when biomarkers’ levels (e.g., circulating concentrations of a metabolite of the chemical exposure of interest) are used as exposure proxy. Since the metabolite’s levels could be influenced by metabolic disorders, which may also influence the health outcome of interest, metabolic disorders (or a genetic polymorphism influencing the metabolite level, e.g. the polymorphism in a gene coding for an enzyme metabolizing xenobiotics) may act as a confounder. No such bias would occur if an external measure of exposure (not influenced by metabolic disorders) had been used as a proxy for exposure. See also [94] for other examples

*Exposure assessment tools*: When designing the approach to assess exposure (including the biological matrix), a key issue relates to the fact that the so-called exposure biomarkers actually reflect levels circulating in the body (if assessed in blood) or excreted (in the case of urine-based biomarkers) concentrations. Thus, as discussed above in they are, in the best case, only proxies of exposure (in the strict sense of the amount of the compound with which the body has been put in contact), and they are not a perfect estimate of the dose at the target organ either. Between the true exposure and these exposure biomarkers lie a series of toxicokinetic processes that vary with time, development stage and generally with many individual behaviours (e.g., physical activity, food intake), and characteristics that may be associated with other exposures or with disease risk (e.g., genetic polymorphisms or body mass index, BMI). As one goes from ambient/external models or data to exposure dosimeters and exposure biomarkers, the measurement error may decrease. Simultaneously, the potential for confounding by individual behaviours or characteristics increases [91] (Fig. 7)– so, there is a trade-off between measurement error and confounding bias from personal factors. As an illustration, in Helix project, persistent organic pollutants (POP) levels in serum were associated with lower BMI in a cross-sectional analysis [92]. However, POPs store in fat tissue, and their blood levels may be influenced by the amount of body fat and by periods of weight gain or loss [46]; such a cross-sectional analysis cannot distinguish between the hypothesis that the POPs influenced the BMI and the hypothesis of an influence of BMI on POPs levels. Good knowledge of the toxicokinetics of the exposure are needed to make appropriate choices, and longitudinal analyses can provide better answers than cross-sectional studies. When toxicokinetic models and detailed personal information on personal factors influencing the toxicokinetics are available, reverse dosimetry (or dose reconstruction via PBPK modelling), allowing to provide an estimate of exposure from circulating or excreted levels, is worth considering [93]. Applying such reverse dosimetry approaches for a large number of exposures simultaneously is cumbersome, and limited by the fact that PBPK models are not available for a large number of exposures and age groups (Cherfan S et al., manuscript).

**Fig. 8:**
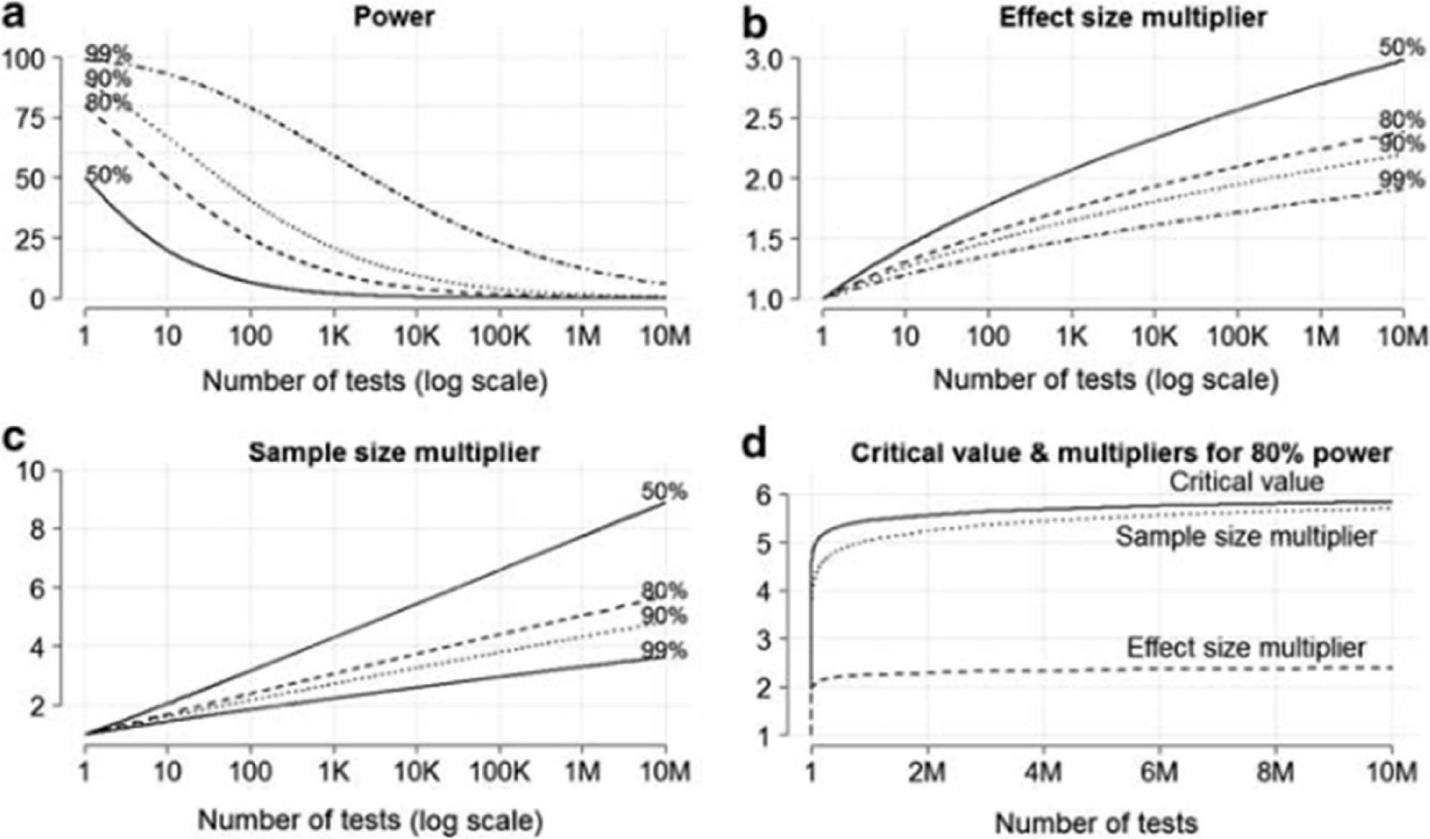
Issues related to power in a context of a large number of tests, under the (conservative) approach of a Bonferroni adjustment for multiple testing. **a** Statistical power as a function of the number of independent tests performed; **b** Effect size multiplier (by which the initial size of the effect of the exposure needs to be multiplied to maintain the power obtained in the case of a single exposure). **c** Sample size multiplier (vertical axis) to maintain the power at the indicated level. Note here the approximatively log-linear association, corresponding to a slope of 1 + 0.68xlog_10_H for a power of 80%, where H is the number of tests. **d** Change in the critical value and multipliers. From Lazzeroni and Ray [65]

*Sample size*: Exposome studies allow to explicitly control for multiple testing (see Fig. 8). Note that issues related to multiple testing also exist in single exposure analyses of cohorts, although less visibly (see discussion) and that not all statistical approaches to analyse etiologic exposome studies imply such correction for multiple testing (see below). To maintain a power of 80% for a single test when 100 tests are actually performed in the study, with multiple comparison correction techniques such as Bonferroni, then the sample size required should approximately double, compared to the situation of a single test [65]. More generally, sample size requirements tend to vary as the log_10_ of the number of tests done (see Fig. 8; this ignores any correlation between exposures).

Moreover, in the context of a study with a sample size large enough to adjust for all exposures through a regression model in order to control for co-exposure confounding, further increases in sample size are needed: for linear and logistic regression models, the required sample size in a model with covariates is k times larger than in a crude model, where k = 1/(1-R^2^) and R^2^ is the percent of variation in the *exposure* of interest that is explained by the other covariates (e.g., other exposures) in the model [95]. This may lead to important increases in required sample size, especially if one aims to disentangle associations between exposures within the same exposure family. For example, in the extreme case of having two highly correlated air pollutants, R^2^ could reach values as high as 0.9, implying that sample size needs to be 10 times larger than in single-exposure studies.

When more specific modelling techniques are used (e.g., suited to sparse data), then we recommend performing a simulation study before starting collecting data to assess the expected power and validate the study design, and in particular the sample size, as well as the planned analytical approach [2].

Finally, some exposome studies claim to aim studying interactions, which are more difficult to detect than main effects. For interactions with an amplitude between a third to a fifth of the main effect, the sample size needed to detect such interaction can be 50 times higher than the sample size needed to detect the main effects [96]. This implies that only very large exposome studies can claim being able to identify synergy between exposures (see below).

As a result of all these facts, and considering that many single-exposure studies are probably underpowered given the “low” exposure levels currently encountered in many Northern countries, often resulting in rather mild increases in disease risk associated with each exposure, exposome studies with a sample size below tens of thousands of subjects may be severely underpowered.

The points stated above did not consider measurement error, which can lead to further increases in the required sample size, or in the number of biospecimens or repetitions in the assessment of exposure in each subject. Simulation studies informed on the within- and between-subject variability of each exposure are a good option to optimize the number of subjects and number of repeated measures. A simulation study in the case of a single exposure with an ICC of 0.2 for example illustrated that, compared to a study of 1000 subjects with a spot assessment of exposure per subject, doubling the sample size is not beneficial in terms of decrease in the attenuation bias. In contrast, doubling the number of biospecimens per subject without increasing sample size would reduce bias. Both options (2000 subjects with a spot exposure assessment or 1000 subjects with two assessments of exposure per subject) provided a similar statistical power [68]. In addition to increasing the number of study participants, the options to increase power include:

1) the reliance on agnostic approaches tailored for sparse (high dimension) data [2, 66];

2) the reliance on external information to reduce the dimension of the exposures considered in an informed manner. This may correspond to toxicological information, allowing for example to group exposures supposed to act on the same biological target [97]; this may also be achieved by relying on a biological marker specific of a given pathway as exposure metric instead of a large number of exposures. For example, a marker of the total xenoestrogenic burden (rather than the multiple exposures contributing to this xenoestrogenic burden) has been tested as a predictor of breast cancer occurrence [103]. This may also correspond to information on sources of exposures allowing to build scores combining exposures, as is done e.g. for the effects of ultra-processed food [98];

3) the reliance on internal information assessed in the study population. For example, high dimension omics markers can also be used to reduce the dimension of exposures, as in the logic of the “meet-in-the-middle” type approaches [99–102].

In conclusion regarding the design of etiologic exposome studies, the strong within-subject variability of many exposures (most of the currently marketed chemicals, most physical, behavioural, psychosocial and infectious factors) makes it important for etiologic exposome studies to rely on a prospective design, which clearly implies to favour the cohort approach over the case-controls design (unless it is nested in a cohort). In this cohort setting, the question of the study design can be seen as an optimization problem with at least four parameters: the number of exposures considered, the number (and timing) of measurements of exposure (e.g., via the use of dosimeters or biospecimens collection) in each subject, the number of subjects, and the duration of follow-up. The two first parameters (number of exposures and of measurements of each exposure) drive the amount of exposure measurement error (which tends to increase – although not inevitably – with the number of exposures, and to decrease with the number of exposure measurement per subject) and the ability to control for confounding by co-exposures, while the two last ones (number of subjects, follow-up duration) condition the number of disease cases and hence statistical power. The novelty of the exposome concept has to some extent led researchers to put emphasis on the number of exposures assessed in a given study and the development of new analytical approaches to assess exposures (not to mention the temptation to simultaneously assess various ‘omics layers), possibly at the cost of the consideration of power and other essential and more conventional parameters. This limited consideration of the number of measurements per subject, of the number of subjects and duration of follow up may also be related to the fact that many early exposome studies were based on existing cohorts and that study cost tends to increase with each of these parameters so that up to now, for a given budget, the sample size and number of measurements per subject tended to decrease as the number of considered exposures increased. This is the opposite of what should happen to limit bias and maximize statistical power.

### Association analyses relating the exposome to biological parameters

#### Considering each exposure separately

**Fig. 9:**
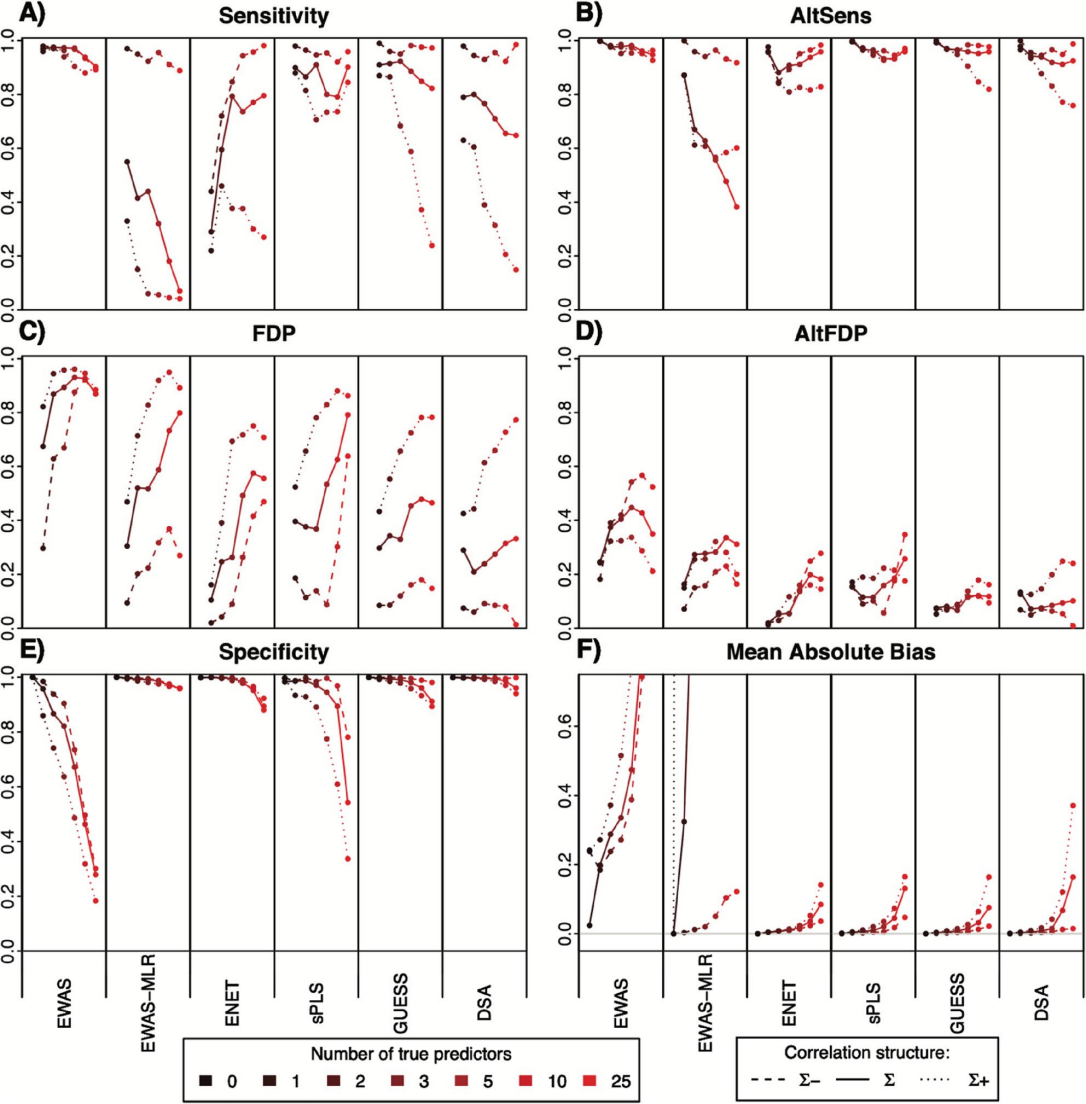
Performances of various statistical methods to identify true predictors of a continuous health parameter, assuming that there are between 0 and 25 true predictors out of 237 exposure variables, and for various levels of correlation within the exposome. Model performances are summarized by **A** their sensitivity, **B** alternative sensitivity (AltSens, see below), **C** false detection proportion (FDP), **D** alternative FDP (AltFDP, see below), **E** specificity and **F** mean absolute bias. For each scenario defined by a number of true predictors varying from 0 to 25, statistics over the 100 runs are summarized by their mean (dot). DSA, Deletion/substitution/addition; ENET, elastic net; EWAS, environment-wide association study; EWAS-MLR, EWAS-multiple linear regression; GUESS, Graphical Unit Evolutionary Stochastic Search; sPLS, sparse partial least squares (from [2]). AltSens is an alternative definition of sensitivity that considered that picking up an exposure highly correlated with a true predictor is almost as good as picking up this true predictor. This indicator is estimated by counting the true predictors identified by the model and counting those not identified by the model using a value below one corresponding to the highest correlation between the true predictor and all exposures identified as hits by the model (see [2]). AltFDP is, similarly, an alternative definition of the false discovery proportion that takes into account the fact that identifying as a hit a variable that is not a true predictor but that is strongly correlated with a true predictor is not as bad a identifying as a hit a variable not strongly correlated with any true predictor (see [2]). The correlation structure either corresponded to that observed in Helix cohorts (continuous line; here 83% of pairwise correlations between pairs of exposures were below 0.2 in absolute value, but 78% of the exposures were correlated at a level above 0.6 with at least one other exposure.), to lower correlation levels (∑-,---- -) or higher correlations (∑ +,^....^) [2]

The first approach used to analyse exposome data corresponded to exposome-wide association analyses (ExWAS), in a transposition of genome-wide association studies (GWAS) [15]. In this analysis, each exposure is regressed separately against the biological outcome, adjusting for confounders, and the resulting p-values are corrected for multiple testing before selecting the hits (the exposures with a p-value below the corrected significance threshold). ExWAS have the advantage of allowing to tailor adjustment factors to each exposure, which is theoretically relevant, as the list of potential confounders depends on the exposure and outcome considered [70] and may thus differ across exposures. This is in principle relevant in the context of the above-mentioned breadth vs. depth trade-off (Table 4). In practice, however, most former ExWAS studies used a unique list of adjustment factors for all exposures. ExWAS have two limitations: 1) They do not control for confounding by co-exposures, as exposures are handled separately (we discuss below approaches simultaneously considering multiple exposures). 2) Relatedly, with a realistic level of correlations between exposures (such as those observed in the Helix project, corresponding to a median absolute pairwise correlation among true predictors below 0.15 [2]), ExWAS can lead to a very high number of false positive associations – higher than 90% of all detected associations [2]. Note that this occurs because of the (relatively low) correlation between exposures, in spite of multiple testing correction procedures. Indeed, multiple testing correction techniques may not be suited to the exposome setting. Multiple testing correction approaches aim to control the family-wise error rate (FWER, the probability of erroneously rejecting even one of the true null hypotheses, that is, to conclude that at least one exposure is associated with health while in fact none truly is), as in the case of Bonferroni approach or, more relevantly in an exposome context, to control the false detection rate (FDR, the proportion of erroneous rejections among all rejected null hypotheses[107]), as in the case of Benjamini–Hochberg approach [108]. These approaches [108] hypothesize that the multiple tests are unbiased. In particular, in the context of genomic studies, they assume that the association between a predictor such as a single nucleotide polymorphism (SNP) and disease occurrence is well estimated, and that it is not necessary to adjust the models for other genetic variables (or SNPs), as the other SNPs are not considered confounders of the association. Yet, in exposome studies, the remaining exposures can confound the association between a given exposure and the health outcome. Consequently, analyses not adjusting for co-exposures are biased and the multiple testing correction methods are not valid. Note that this also applies to the multiple testing correction procedures developed for dependent tests, such as the false discovery rate control procedure from Benjamini and Yekutieli [107]. This latter test is valid when studying the possible effects of a single factor on multiple diseases, or of multiple treatments on a single disease in the context of a randomized study in which different groups of subjects receive different treatments, avoiding any confounding bias. It is not suited for a study in which the associations of multiple and possibly correlated exposures assessed in a single population group are estimated in relation to a given health outcome. Coherently, in the simulation study by Agier et al. [2], ExWAS had a high false positive probability, with about four hits out of five that did not correspond to true predictors in the tested setting. ExWAS also tended to have the lowest specificity and the largest mean bias in the estimated association among all compared models when the number of true exposures increased (see results reproduced Fig. 9). The sensitivity of this model was high, which was of limited interest in a context of a very high false detection rate (i.e., in which this model tended to conclude that a very large proportion of exposures were true hits, which they were not). The false detection rate increased with the number of true predictors and with the correlation level between exposures (Fig. 9), illustrating the fact that confounding by co-exposures is the mechanism underlying the inability of ExWAS to control the false detection rate in spite of the application of a multiple testing correction procedure.

**Table 4:** Issues identified in exposome studies, with their possible consequences and suggestions of cures

Issue and consequences	Possible cures
*Exposure assessment*	
Increasing the coverage of the exposome (i.e., the number of exposures assessed) is expected to decrease power and increase false positive rate (if sample size remains constant)	Increase sample size [65]
Within-subject variability of exposure	Assess (and possibly average) exposure repeatedly during the a priori relevant time window (which may be exposure-specific) [67]
Differential measurement error in exposures, leading to different statistical power to detect associations with distinct exposures	Tailor exposure assessment to each exposure (e.g., increase the number of measurements per subject for exposures with the highest temporal variability)
Causal relations within the exposome (e.g., if green space, air pollution and a marker of oxidative stress are simultaneously considered as being part of the exposome)	The modelling should take these relations into account. Draw DAG of assumed causal relations and adapt modelling accordingly, or only consider exposures not influencing each other
*Issues related to confounding*	
Correlation within the exposome and confounding by co-exposures. Expected to strongly influence sensitivity and false positive rate	Rely on multiple regression models adapted to sparse data [2, 66]
Confounders may differ for each exposure	Define exposure-specific adjustment factors (if the analytical approach can accommodate this); perform sensitivity analyses with various sets of adjustment factors
*Curse of dimension, limited power*	Increase sample size [65]; reduce exposome dimension using toxicological knowledge [97, 103]; rely on statistical models adapted to sparse data [2, 66]
*Statistical modelling*	
“Depth vs. breadth” trade-off (less flexible modelling, e.g. in terms of adjustment factors or shape of exposure response functions as the number of exposures considered increases; more limited ability to investigate exposure-specific biological mechanisms of action)	*Adjustment factors:* Correcting exposures for their association with potential confounders in a preliminary modelling stage, and using these corrected exposures in the (stage 2) health model is an option. *Shape of ERFs:* Include transformed forms of exposure variables—e.g., fractional polynomials [104] (this is at the cost of an increase in the dimension of the problem). *Mechanisms:* Study biological mechanisms in single exposure settings (and possibly in toxicological approaches, relying on human studies only at a confirmatory step)
Model instability related to the chosen modelling approach, if it includes some random process (e.g., for Lasso-type approaches)	Consider approaches to stabilize model, keeping in mind it will affect its performances [105]
Variability in results related to the multiplicity of the modelling options [106]	Assess the “vibration of effects” [106] associated with the diversity in modelling options, including choice of adjustment factors and type of statistical model

*DAG* Directed Acyclic Graph. *ERF* Exposure response functions

All in all, we believe that the very high rate of false positive hits expected from ExWAS studies should be seen as a major drawback and recommend to only consider ExWAS analyses as a report of crude associations and not as the final study results. If one sets the bar lower by considering that a model performs well if it captures the truly-associated exposures or exposures that are highly correlated with them, then the false positive rate of ExWAS decreases substantially, but other methods can still do better (see “AltFDP”, Fig. 9). Note that capturing an exposure highly correlated with the truly-associated one is only useful if such variable carries information on the nature of causality (e.g., it identifies the family of chemicals or the behaviour causally linked with the disease). High correlations between exposures can be observed for other reasons than the exposures belonging to the same chemical family or being associated with the same behaviour.

#### Simultaneous consideration of multiple exposures

A first simple option simultaneously (and not successively) considering multiple exposures consists in adjusting for all exposures simultaneously in a single (“classical”) regression model. The requirement of having a number of disease cases (and non-cases) of at least five to ten times the number of covariates to limit bias in estimates and avoid convergence issues and low power [109, 110], implies that a study using this analytical approach with one thousand covariates and a binary or survival outcome would on this basis need at least fifty to one hundred thousand subjects (assuming a ratio of about one disease case for ten non-cases).

A second option consists in first considering all exposures separately as in an ExWAS and then conducting analyses mutually adjusted on the exposures most strongly associated with the outcome (in terms of p-value), which constitute likely candidates as confounders. In a simulation study, Agier et al. considered this so-called ExWAS-MLR (ExWAS followed by Multiple Linear Regression) approach, in which in a second step all exposures significantly associated with the outcome in the first univariate step, after correction for multiple testing, were jointly considered in a single multiple regression model. This approach turned out to perform better than ExWAS in terms of false detection proportion and specificity, and less so in terms of bias in the parameters associated with the true predictors and in sensitivity [2] (see Fig. 9).

Approaches more specific to the situation of a rather large number of predictors, sparse data and/or of high correlation among some predictors have been developed in the last couple of decades, including variable selection techniques, penalized regression, or Bayesian approaches [111]. These approaches have been shown to produce a smaller percentage of false positive hits compared to ExWAS and ExWAS-MLR, while possibly maintaining high sensitivity to detect true positive signals [2], depending on sample size. It is important to note that these techniques do not control explicitly to a given, pre-specified false discovery rate (although they do have tuning parameters that can influence sensitivity and false detection rates). In the simulation study by Agier et al. based on the exposome correlation structure observed in Helix project, the method that provided the lowest (but nonetheless high, 34% on average) false detection proportion was the Deletion-Substitution-Addition (DSA) algorithm [2]. When considering that the model performed well if it captured the truly associated exposures or the ones that were highly correlated with them, the (alternative) false discovery rate of DSA was below 10% in the simulation study. Some recent developments are trying to estimate and control the false discovery rate in the context of variable selection [112, 113].

Another way to facilitate the estimation of associations between exposures and health outcomes is to incorporate into the model some structure, based on pre-existing knowledge. Examples include imposing that all exposures from the same exposure family are expected to have a similar association (which is a strong assumption given that chemical function/family is generally not enough to predict biological mechanisms), or, when studying polychlorinated biphenyls, assuming that the magnitude of the effect of each compound is determined by its degree of chlorination (such assumptions may be made more relevant in the future by developments in predictive toxicology). This is mostly done using Bayesian techniques, although frequentist approaches are also possible. Structure can be further imposed by conducting an outcome-wide analysis (if data allow, which is often the case in a cohort setting, in which multiple outcomes are simultaneously assessed), an approach that can identify exposures with multiple effects on health. In that framework, one can choose techniques that assume, for example, similar effects of the exposures on different outcomes, with potential gains in statistical power [114]. Other examples include techniques that use intermediary biological layers (such as epigenetic data) to improve the selection of associated variables. These techniques mentioned above, related to the meet-in-the-middle concept, can help lowering the number of false positives under some causal structures [100].

Other approaches aim at reducing the dimensionality of the exposome by creating weighted combinations of exposures, either defined a priori (e.g. sum of polychlorinated biphenyls (PCB) congeners) or using principle component analysis (PCA), partial least squares (PLS) or similar techniques, or by grouping subjects (clusters) sharing similar exposomes [52, 115]. These new derived variables can be obtained using or not using information on the health outcome (supervised and unsupervised learning, respectively). All these techniques can then provide estimates of the association between the derived variables and the outcome. A concern with unsupervised learning is that some of the criteria used to combine exposures (such as correlation between exposures) are unlikely to be predictive of an effect on the health outcome considered: it is not because two exposures are correlated (e.g., because they are related to a same given source or behaviour) that they act on the same biological target.

### Association analyses – non additive, synergetic and supra-linear effects

Other interrelated issues that can be addressed in etiologic exposome studies are the existence of interactions and of other “mixtures” effects (note that in humans, mixture studies typically refer to studies considering a rather limited number of exposures, typically 5 to 50, with a rather high correlation level, and for which the question may be either to single out bad actors, or to identify if the mixture as a whole has a deleterious biological effect). Toxicological studies in animals are probably the most efficient setting for such questions. They considered mixtures of typically 5–20 chemicals using approaches allowing to avoid the correlation between exposures limiting observational studies [116, 117]. In vitro toxicology can consider mixtures of more than a hundred chemicals [118]. In observational studies in humans, various analytical options exist. Some methods deal with the combined effect of exposures by providing some sort of association between the mixture of exposures and the health outcome, but not really assuming the presence of interactions. This is the case of techniques like weighted quantile sum (WQS) regression [119]. One of the ideas behind this is to try to capture the joint effect of increases in many exposures, as individuals may be exposed to many of them at the same time. Only testing the effect of the mixture, and not that of the individual exposures alleviates multiple testing issues. If one wants to consider whether, when two exposures are present, the effects are stronger than the addition of the individual effects (synergy), one needs to test for (some form of) interaction. This should be done keeping in mind that statistical interactions are not systematic signs of toxicological synergy, in particular outside an additive (risk difference) framework [5]. As mentioned above, the detection of interactions requires much greater sample sizes that when exploring main effects only. In an exposome context, considering interactions may dramatically increase the search space, compounding dimensionality issues, and the number of tests. For example, when considering 200 exposures, the number of potential order two interactions is 19,000. Still, some techniques are especially tailored to search for interactions in high dimension settings and may outperform more traditional techniques [4]. Interactions can actually be difficult to distinguish from non-linear effects (e.g., the product of two highly correlated exposures, X_1_ x X_2_, used to capture interactions, can be very close to X_1_ x X_1_ = X_1_^2^, used to capture nonlinear effects). Some techniques, like Bayesian Kernel Machine Regression (BKMR), allow the estimation of complex high-dimensional surfaces that can capture non-linear effects and complex interactions. These can be used to explore the effects of mixtures of variables [120]. Note that the approaches discussed in this paragraph are, at present, mostly limited to cases with a small number of exposures. However, extensions of some of the techniques (e.g. WQS) have already been applied to cases with more than 800 exposures [121]. Further extensions will probably continue to be developed, which will likely widen the applicability of these techniques.

Several review papers provide more details on potential statistical techniques that can be used to conduct associations analysis between the exposome (or “mixtures) and health outcomes, including the ones belonging to the main groups of techniques discussed here [122–124].

One topic that has been little explored is the analysis of longitudinal exposome data, in the sense of using repeated measures of the exposome and/or the health outcome. A longitudinal structure introduces further complexity as many different hypotheses can be tested, the dimension of the data escalates further, and many different analysis techniques can be used for modelling. A recent study compared different analyses strategies in a restricted case where repeated exposome data are related to a health outcome measured at a single point in time [125]. The paper illustrated some of the challenges in the analyses of such data, and that the performance of different analysis methods may depend on the specific scenario being considered. The methods mentioned above for outcome-wide exposome analyses can also be used to handle longitudinal exposome studies [126].

## Quantifying the health impact of the exposome (risk quantification)

### Principle and challenges of environmental burden of disease studies

Identifying associations or causal links between the exposome and subclinical parameters or health, as done in the etiologic studies presented in the previous section, may not be enough to manage the health risks induced by the exposome. In a context in which many exposures are possibly harmful, society and decision-makers may want to rank exposures in terms of attributed deaths or disability-adjusted life years (DALYs), as provided e.g., in the context of the Global Burden of Disease project [127], in order to inform decisions about which factors to regulate in priority. In addition, a fraction of regulations tend to be risk-based. That is, they rely on information relative to the fact that the exposure factor induces a number of disease cases or deaths that is above or below the level deemed acceptable. Finally, studies on the estimates of the population attributable fraction [128] of disease cases or DALYs attributable to one or several exposures under alternative exposure scenarios can also be used to identify exposure reduction scenarios (or policies) that should be implemented in order to achieve a given health gain in a specific population. This has been illustrated for atmospheric pollutants [129], but could also be done for several exposures simultaneously.

This estimation of the fraction of disease cases attributable to the exposome implies to have knowledge on the diseases possibly induced by each exposure, on the corresponding exposure–response function (provided by etiologic studies), and on the distribution of exposures in the considered population (from descriptive exposome studies as described above). This approach is termed (quantitative) health impact assessment (HIA), or environmental burden of disease assessment (some authors distinguish these expressions, see [130] for a discussion on terminology). Health impact assessment studies considering a large number of exposures, such as those from the Global burden of disease project [127] and studies of the disease burden associated with dietary factors [131, 132] should be seen as exposome (as well as outcome-wide) studies.

Some challenges of environmental disease burden studies include the risk of underestimation bias due to the lack of consideration of specific exposures and their effect because of limited data quality or availability, issues related to causal relations or correlations between some exposures, and more conceptual issues related to the definition of counterfactual scenarios, the notion of attributable disease cases and their estimation [130].

### Study design of exposome burden of disease studies

The design of health impact assessment (or environmental disease burden) studies has been reviewed elsewhere [130, 133] and consists in the following series of tasks (adapted from [130]):

1. Definition/identification of the environmental factors or intervention considered.
2. Definition of the study area and population.
3. Definition of the counterfactual scenarios compared and of the study period.
4. Assessment/description of “exposures” in the study population under each counterfactual scenario.
5. Identification of the hazards (health outcomes) induced by the factors considered, of the corresponding level of evidence and, if available, of dose–response functions, usually from the literature.
6. Assessment of “baseline” (usually, current) disease frequency or of the DALYs attributable to the health outcomes considered, if needed.
7. Quantification of health impact (e.g., in number of disease cases or DALYs) associated with exposures under each scenario.
8. Possibly, quantification of the corresponding social and economic impacts.
9. Uncertainty analysis.

**Fig. 10:**
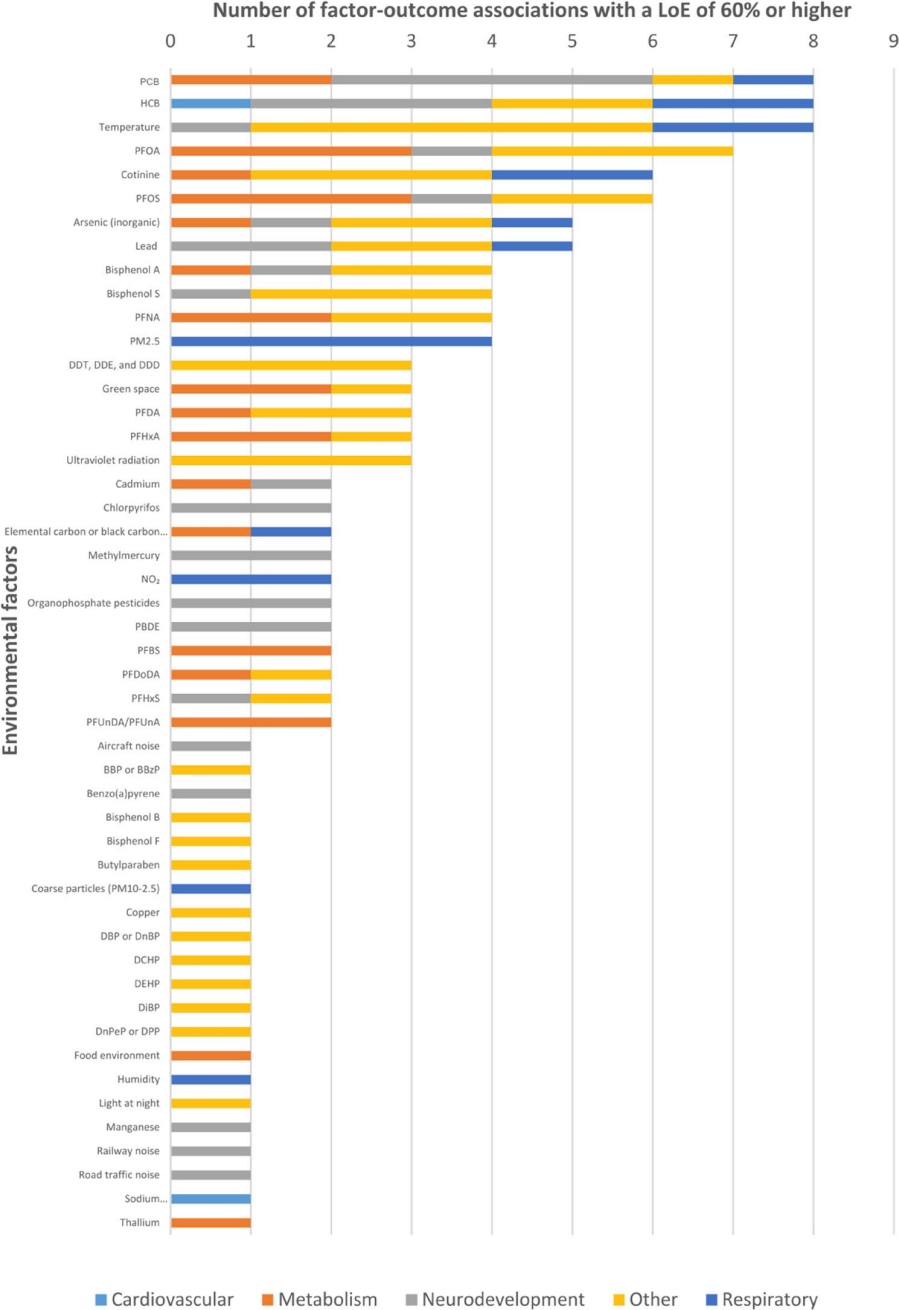
Number of types of children diseases associated with a set of exposures, restricted to those with a likely or very likely level of evidence LoE. The level of evidence was assessed from a review combining epidemiological, toxicological and mechanistic streams of evidence for 611 exposure-health outcome pairs [64]

In addition of possibly being exposome studies, HIAs, which are aggregates of individual studies, can be fed by exposome studies. This is typically the case of the steps 4, 5 and 7 above. Indeed, the step 4 of assessment and description of exposures can rely on an exposome descriptive study (see above) such as a biomonitoring survey or a (representative) cohort study describing a large number of environmental factors. In most past environmental burden of disease studies, multiple sources of information and studies are typically considered at this stage. However, relying on one single exposome study would have the advantage of providing a distribution of the exposures to each considered factor in the study population at a given time point together with estimates of the pairwise correlations between these exposures. These correlations need in principle to be considered at the step of quantification of the impact (see below). The literature-based step 5 of identification of the hazards induced by exposures and of the corresponding level of evidence can also benefit from exposome studies. It is relevant to consider not only human exposome studies, but also human single exposure studies and toxicological as well as mechanistic studies when assessing the level of evidence associated with each exposure-health outcome pair, as typically done by assessments of carcinogenicity by IARC or other studies [64](see Fig. 10). Exposome studies allow in principle to provide exposure–response functions for a large number of exposures simultaneously. These have the advantage of being adjusted for confounding by co-exposures (see above) but, compared to meta-analyses typically used at this step, have the disadvantage of being more prone to random fluctuations because of their lower sample size (a typical trade-off between bias and variance). If no meta-analysis is available, then using an exposure–response function from a (possibly exposome-type) single study is advisable. Indeed, not considering isolated studies when no meta-analysis is available implies to ignore the corresponding association (i.e., to assume a zero risk), leading to a bias if the effect is deemed likely (e.g., based on mechanistic or toxicological evidence). This bias towards the null is possibly stronger than the error induced by relying on a possibly little robust dose–response function based on an isolated study (another case of bias-variance trade-off). This is an illustration of the underestimation bias mentioned above. Coherently with this logic, in a recent review of available dose–response functions regarding a large number of chemical exposures, published meta-analyses were considered but also, when no meta-analysis was available, individual studies [134]. In addition to the lack of available exposure–response function, the underestimation bias may be due to some information regarding one exposure being missing, or the level of evidence regarding its health effects being considered low, or information on the baseline incidence of the disease being of poor quality. In the past, the default option in HIAs was indeed to ignore exposure-outcome pairs whose level of evidence was not considered to be very high. For various reasons discussed elsewhere, including the fact that the level of evidence regarding the effect of an exposure on health more often tends to increase than decrease over time, this will bias towards the null the burden of disease attributed to the exposome [130]. An alternative is to try to include in the assessment, whenever possible, exposure-outcome pairs with a less than certain level of evidence. The corresponding attributable burden can then be weighted by the level of evidence of the exposure–response pair to limit the risk of overestimation bias [130, 135]. Regarding the correlation between exposures, when aggregating impacts from various exposures likely to influence the same disease, the information on the correlation structure of these exposures is also relevant; indeed, the combined impact of two or more exposures is expected to differ between populations in which they are strongly correlated and populations in which they are not correlated [130, 136]. This is true even in the absence of (statistical) interaction in the effects of these exposures, and of course also in the case of interaction. Exposome studies can provide the information on this correlation structure and possibly on the existence of statistical interactions on their effects (as discussed above). This combination across exposures of impacts on a given disease may be done using traditional “population attributable fractions” estimates [130, 136]. Other approaches, such as microsimulation (simulating all individuals in a population and modelling the disease occurrence across time as a function of their exposures) might be more efficient in handling these situations, in addition to providing a straightforward way to estimate the life years lost because of exposures [130, 137]. Relatedly, the causal relations between exposures need to be considered; for example, in an exposome burden of disease study considering both the impacts of greenspace and air pollution exposure, the fact that a part of the health effects of greenspace on health are mediated by changes in exposure to air pollution would need to be accounted for.

The question of the aggregation of exposures in the presentation of the study results is another practically important point, as health impact assessment studies covering a large fraction of the exposome can be used to rank exposures in terms of impact. Here, since it is expected that many factors may have a small impact, presenting this impact exposure by exposure or by groups (e.g., adding the impacts of all substances with endocrine-disrupting properties or acting as neurotoxicants, or presenting them for each factor in isolation) will provide different rankings of the exposures. Such choices regarding the grouping of exposures need to be carefully justified as they can influence any decision that might be taken considering this ranking.

## Interventional aim: Modifying the exposome

### Aim and principle of interventional exposome studies

Health impact assessment studies can tell which exposures should be prioritized to obtain the largest health gains, which health gains could be obtained from pre-specified changes in exposures, or which exposure change to target to obtain a specific decrease in the disease burden. They leave open the question of how to bring about such changes in exposures. This is one of the purposes of interventional studies on the exposome. These studies aim to identify strategies allowing to decrease multiple exposures in a given population, the extent of the decrease achievable, and possibly identify barriers and facilitators to such decreases, as well as the social or psychological mechanisms related with these changes; another aim may be to quantify the change in a biological parameter or health outcome induced by the intervention. They may also inform the sources of exposures to specific factors: by showing if the concentrations of specific chemicals decrease following an intervention on cosmetics or diet, they may provide indirect evidence of the contaminants present in cosmetics or diet (see [138] for an example related to cosmetics).

All types of intervention can in principle be targeted. Interventions at the levels of individuals can, maybe somewhat paradoxically, serve the purpose of demonstrating the need for public action at other levels: if, for example, an intervention on individual changes in food behaviours (e.g., to stop eating canned food) only entails a 10% decrease in the body levels of bisphenols then this can be interpreted as providing an indication that in order to reach strong decreases in human exposures, regulations limiting the concentrations of bisphenols in all milieux rather than actions based on individual motivation, may be needed. Similarly, an intervention that proves to be only efficient in a subgroup of the population (e.g., the most educated people) may be seen as warranting action on the environment, which may impact more homogeneously the whole population. Interventions can exist at the level of individuals (changes in diet [139], transportation mode [140], cosmetics use [138]…), at the level of institutions or on the environment as a whole (from infrastructures to food products offer or transportation services). Interventions should not be considered in a narrow sense and can rely on a diversity of instruments (information/education, provisions and infrastructures, subsidies and gifts, taxes, command-and-control type decisions…). Consequently, such studies can rely on multiple disciplines from epidemiology to behavioural and cognitive science to economics, political sciences and law. Regarding the induction of changes in exposures mediated by behavioural changes, the area has benefited from strong inputs from behavioural sciences and psychology, that have proposed various models of behavioural changes (see e.g., [141]). As an illustration, according to COM-B model, behaviours (B) are inducted by capabilities, opportunities and motivations (COM), which can in turn be influenced by various intervention functions (training, coercion, incentivisation…) and policy categories (regulation, service provision, environmental/social planning…), as summarized in the “behavioural change wheel” [141].

### Design- and analysis of interventional exposome studies

A central design option relates to the possibly randomized nature of the study. If the study is randomized, which is generally preferable to limit bias, the question of the randomization unit should be solved considering the type of intervention and the possibility of spill-over effects. An intervention at the level of the environment generally implies a randomisation of spatial units or communities and not individuals. When randomization is done at the group level, options include (conventional) parallel clusters (in which each cluster remains in the same status with regards to the intervention throughout the whole study) or a stepped-wedge design (in which the intervention is implemented in all clusters at various times, so that there is a before and an after-intervention observation period in each cluster) [142]. The stepped-wedge design is generally favoured over the parallel design in cluster trials with a strong intra-cluster correlation [142].

The sample size should be estimated considering the design, the expected fraction of subjects lost to follow-up, the distribution of the parameter (exposure, biological variable or health outcome) of interest, the number of exposures considered (entailing a possible need to correct for multiple testing, and hence to increase sample size, compared to a study with a single primary outcome) and the targeted changes. The required sample size is generally larger, in a given context, when targeting a change in a health rather than in an exposure parameter, given the possibly weaker, longer-term and more complex association between behaviours and biological parameters than between environmental levels, behaviours and personal exposures. Follow-up will possibly be longer in studies also considering changes in biological parameters than in studies only aiming to identify changes in exposures and their causes, although assessing whether behavioural changes are sustained on the mid- or long-term may be of interest. Clustered interventions require specific considerations related to the number of clusters, the within-cluster correlation and possible variations in cluster sizes, and should generally aim towards increasing the number of clusters rather than the size of each cluster [143]. The targeted exposures should be defined considering the intervention (e.g., the chemicals possibly present in cosmetics if the intervention modifies the use of cosmetics). The duration of exposure assessment or number of biospecimens collected within each subject and time-period should possibly be increased to limit the impact of within-subject variability in exposure, as mentioned above.

## Discussion

We have summarized issues related to exposome studies aiming at describing the exposome in human populations (*descriptive aim*), assessing its possible effects on biological (subclinical and clinical) parameters (*etiologic aim*), quantifying the corresponding health impact (or the expected health impact of any specific change in a group of exposures; *risk assessment* aim) and finally at modifying the exposome (*intervention aim*). Although exposome studies have originally been conceptualized as focusing on only some of these four aims, in particular the etiologic aim, we believe that it is important to realize that the four types of studies discussed are in fact “exposome” studies and that more consistency and dialogue between these types of studies and the associated disciplines should be sought.

Our general assessment is that exposome studies already have the ability to provide and do provide descriptions of (parts of) the exposome, and of the associations between the exposome and socio-demographic and geographic characteristics (descriptive aim, environmental justice studies); that there has been progress in exposome-wide HIA (or risk assessment, or environmental burden of disease) studies, for which a rigorous framework and relevant methodologies exist, although these are limited by the lack of robust dose–response studies for many relevant exposure–response pairs. When it comes to aetiology, with very few exceptions [144], prospective studies simultaneously considering hundreds of exposures had a sample size of a few hundred to about a thousand subjects which, even for continuous outcomes, is probably underpowered; the ExWAS modelling is moreover prone to a high rate of false positive hits [2] (which might partly explain its use in a context of underpowered studies…). We believe that much larger prospective studies with a number of participants around a hundred thousand individuals, and possibly more if thousands of exposures are assessed, and accurate assessment of exposures, are warranted to overcome this issue of low sensitivity, as well as reliance on statistical approaches adapted to sparse data. The strong increase in sensitivity of chromatography and mass spectrometry over the last decades now makes it possible to quantify hundreds and possibly thousands of chemical contaminants or their metabolites in urine and blood samples of a few millilitres [24, 25, 118]. Such an active follow up of cohorts of hundreds of thousands of volunteers is possible, as numerous examples worldwide demonstrate [145]. As a consequence, the main obstacle for such large scale exposome studies is probably that of securing the required budget, in the tens or hundreds of million Euros for each cohort.

We did not systematically discuss toxicological studies, but pointed to their essential contribution to exposome research, in particular when it comes to the etiological aim (both for the identification of the underlying biological mechanisms – which corresponds to toxicodynamics – and hazard identification on an exposure-by-exposure basis and in the context of mixtures), for risk assessment (at the step of assessing the level of evidence associated with each possible exposure-outcome pair [64]), and also for the descriptive aim, in particular to describe within-subject variability in exposure, which can be informed by toxicokinetic studies. We did not either specifically discuss “mechanistic” or “cross-omics” studies [146] aiming at unravelling associations between the exposome, multiple ‘omics layers and health in order to highlight mechanisms of action of pollutants (with the exception of the case when they consider a single pre-identified biological parameter, such as the initiating event of an adverse outcome pathway, which corresponds to the single outcome case we presented). In these studies considering multiple biological parameters, the issues associated to confounding by co-exposures, to within-subject variability (since many ‘omics signals tend to vary quickly within-subject [147], just like many exposures) and to low power identified for single outcome exposome etiologic studies also exist. Their impact is probably larger than in single outcome exposome studies, given the higher dimension of multi ‘omics data, and are compounded by a less rigorous framework for causal inference due to the complex causal relations between these multiple layers [148, 149]. As a consequence, robust results on these issues may come later and at a higher cost than for etiologic exposome studies focusing on a single biological outcome, and in vitro and in vivo pharmacodynamic studies may remain the main source of knowledge on such mechanistic issues.

We discuss below issues related to the definition of the exposome, to study design, to the connexion between exposome and “mixture” studies on the one hand and exposome and single exposure studies on the other hand. We conclude with considerations on possible ways to address the aims assigned to exposome studies.

### The exposome(s)

The original definition of the exposome encompasses all environmental factors, including lifestyle factors [6]. Since the environment of humans is what surrounds them, this can be interpreted as including physical, chemical, biological factors of external origin (in particular infectious factors) and social-behavioural factors. This definition explicitly includes lifestyle factors (diet composition, passive smoking, cigarette smoking), aesthetic factors (that can be seen as being physical or psychosocial in nature) and systemic factors such as climate change (including physical factors such as temperature and extreme weather events, biological factors such as changes in exposure to infectious factors…), biodiversity loss (related to biological factors and their consequences on the chemical and physical environment), antimicrobial resistance. Of course, a specific domain could also be used for systemic factors (Table 2). Another exposome definition presents the exposome as “an integrated compilation of all physical, chemical, biological, and (psycho)social influences that impact biology" [31]. This definition may read as if only factors known to influence biology should be a priori considered to be part of the exposome, what we see as too restrictive and likely to limit the discovery of new factors influencing biology and health. Sometimes an “internal exposome” is defined and factors such as the “inherent metabolic and cellular activity” are encompassed into the exposome [27]. It has also been claimed that it would be reasonable “to consider the “environment” as the body’s internal chemical environment and “exposures” as the amounts of biologically active chemicals in this internal environment” [150]. An issue with this logic is that a large number of endogenous molecules can be influenced by the exposome without being “signatures” of it (in the meaning of something specific to it and not shared with any other factor). In our view, although of course biological matrices internal to the body can be used to describe the exposome (e.g., via the levels of exposure biomarkers that have a good sensitivity and specificity with respect to exposures) and of course its impact on biology and health (via so-called biomarkers of effect), not all internal parameters should be seen as being part of the exposome. For example, CRP (C-reactive protein) levels or concentrations of immunological, inflammatory markers or markers of oxidative stress, which may also be influenced by diseases, aging and by the genome, should not be seen as being part of the exposome. Although interesting from a mechanistic point of view and relevant scientifically if the sample size and other aspects of the study design allow to make use of the information that would be thus collected, we would advocate for the endogenous molecules or biological parameters not to be seen as part of the exposome, with the exception of exposure biomarkers in the strict sense. This is consistent with the genome definition, which does not include all possible downstream products of the genes such as protein levels or metabolic markers.

So far, to our knowledge, most so-called exposome studies are quite far from assessing efficiently all the components of the exposome; for example, although studies assessing tens or hundreds of chemical exposures (which may very roughly correspond to less than 0.1–1% of the chemical exposome, if we only consider the possibly 350,000 chemicals on the market [151], thus ignoring those naturally occurring or banned but persistent) exist, while other studies characterized diet [36] and other lifestyle factors and other exposures to a variety of biological factors including bacteria and viruses (e.g., studies on the gut microbiota and its effects [152]), we are not aware of published studies that managed to simultaneously capture a large proportion of the chemical, social-behavioural and biological factors. Schematically, the field currently includes studies in which up to about a hundred of exposures from diverse exposure families have been assessed (as was done in HELIX [45] or LIFE projects [79]), and much larger studies which either considered a very limited number of exposures (e.g., only air pollution, temperature and ZIP code as a proxy of other exposures), or were of purely cross-sectional nature (such as those relying on biomonitoring surveys such as most analyses of NHANES studies [15]), limiting the relevance for time varying exposures. One exception corresponds to a prospective study by Patel et al. on mortality based on NHANES population, in which socio-behavioural (such as socio-economic status, alcohol or tobacco use, physical activity), biological (hepatitis A or B antibodies) and chemical (such as PCBs, phthalates and also nutrients) factors had been assessed [144].

#### Number of subjects and of exposure measurement points per subject

In his seminal 2005 paper, Wild discussed the development of studies on the genome assessing an increasing number of genetic factors. He mentioned that “The consequence may not be greater clarity but a greater number of chance findings and an increasing difficulty of dealing with the sheer volume of data in the absence of parallel advances in data analysis. Things may get worse before they get better” [6]. In a way, this prophecy can also be applied to the exposome, because of the possible over-emphasis on the number of exposures assessed over the more conventional other parameters driving statistical power such as sample size in the early exposome studies.

The need for large funding allowing the sample size of cohorts to increase together with the number of exposures considered and with the number of exposure assessment points per subject (via biospecimens, questionnaires and dosimeters) calls for an increase in the funds dedicated to exposome research. This could enable large cohorts in which phenotypes, behaviours and large numbers of exposures are accurately characterized, with repeated sampling of biospecimens being collected and stored. It is striking that, in spite of important efforts in favour of exposome research, the EU, which had made such effort in the 1990 s via the EPIC project, did not support yet the launch of a well standardized EU-wide exposome cohort. Such a need has been recognized in a recent report to the European Parliament [153].

#### Mixtures *versus* exposome research

Large bodies of literature in toxicology and epidemiology refer to *mixtures*. In toxicology, this corresponds to a well-defined area in which the effects of several exposures (typically, between two and two dozens) on a given biological endpoint are assessed in a systematic controlled approach. These studies schematically allow to test each exposure separately as well as combinations of all exposures for a wide exposure range and avoiding correlations between exposures and their consequences. A frequent aim of such *mixture toxicology* studies is the identification of departure from additivity (the study of synergy) [116]. In epidemiology, the term *mixture* is generally used for studies considering a rather limited number (again, rarely more than two dozens) of sometimes highly correlated exposures, such as atmospheric pollutants or chemicals from a few families. The key issues in these epidemiological studies may be the identification of the “bad actors” within the considered mixture (e.g., do PM_2.5_ and NO_2_ have independent effects on mortality?) and possibly the identification of a biological effects of the mixture as a whole, without generally allowing to answer if any mixture effect is different (e.g., larger) than that expected from the individual effects of its constituents (synergy or antagonism). Indeed, if the observed exposures are strongly correlated in humans, this question is more easily tackled in the controlled setting of toxicology, in which the effect of each exposure can easily be tested independently from the other exposures. These animal studies do not allow providing exposure response functions valid in humans, so that their relevance for the impact assessment aim that we discussed is more limited (mainly, to inform the level of evidence regarding a possible effect; they can also be used in safety assessment, following a logic of “margins of exposure” [154] to identify if a population is in a safe exposure range, or hazard quotients, to point the bad actors in a mixture [155]).

#### Relevance of exposome studies for the study of synergy between exposures

Exposome studies are sometimes put forward as a way to identify synergy between exposures. Sometimes the expression of “cocktail effects” is used, without this expression being always clearly defined – it could either refer to the cumulative effects of exposures or to biological interactions, or both. Our view is that unless very large sample sizes are achieved (typically beyond 10^5^ subjects with very accurately characterized exposures) or very strong a priori hypotheses regarding the interactions to consider are a priori available, limiting the number of interactions to test (e.g., as a result of a toxicological or molecular study [156]), current conventional exposome studies are unlikely to discover true synergy between exposures. Human exposome studies without a very large sample size are probably more efficient to confirm a priori hypotheses on synergy generated by other approaches than to generate hypotheses on such synergy. In addition, toxicologists and epidemiologists tend to have different views on synergy, and, as already discussed, epidemiologists have long conflated synergy with statistical interactions [5, 157]. Finally, it should be reminded that the toxicological literature, while having identified a few situations of biological interactions with plausible underlying mechanisms (such as those involving grapefruit juice or compounds able to form a supramolecular ligand [156]), is not in favour of synergy being frequent [116]. Consequently, the question of the study of synergism may have been given overdue importance. This should not minor the relevance of the question of the *cumulative* effects of exposures (i.e., doses and impacts adding up without synergy), which is relevantly addressed in health impact assessment studies, as we reviewed above.

#### Exposome *versus* single exposure studies

As discussed here, several of the limitations of single exposure studies can be efficiently tackled by exposome-type studies; for other limitations, exposome studies may not be generally more efficient than single exposure studies (see Table 1 for a summary), while exposome studies also have additional specific challenges (summarized Table 4).

Exposome studies have the clear and important advantage to limit the risk of publication bias, both at the manuscript preparation and publication stages, as they allow showing associations of all considered exposures with the outcome(s) of interest. They tend to have a higher throughput than single-exposure studies (disregarding the crucial step of leveraging the study funding), at the cost of generally being less able to go “in depth”.

The challenges of exposome studies (summarized in Table 4) include participation burden and the associated possible selection bias. This issue also exists for “single exposure” cohorts in general actually, but possibly to a lesser extent, depending on the approaches used to assess exposures. As discussed above, the correlation between exposures induces confounding bias in ExWAS-type analyses, and also probably affects the efficiency of alternative statistical approaches developed for sparse data [2]. Note that, although little recognized to our knowledge, this correlation can also be an issue in single-exposure studies. Indeed, single exposure studies can be seen as one step of an ExWAS analysis (not corrected for multiple testing and possibly not for confounding by co-exposures): statistically, there is no difference between a) the results from a study team conducting and publishing successive studies on a series of exposures assessed in the same cohort, and reporting the effect estimates associated with a given outcome separately for each exposure in different publications, and b) the study team analysing the associations between all these exposures and the outcome in this same cohort and reporting the results in a single publication, once all exposure data are available, using an ExWAS approach not corrected for multiple testing. This approach has been shown to suffer from a high false positive rate, given the (albeit rather weak) correlation within the exposome [2]. In this line, the limitations of the ExWAS approach that we discussed should not be seen as specific to exposome studies assessed with ExWAS but also possibly applies to successive single-exposure studies published relying on the same cohort. The advantage of exposome studies over single exposure studies is that, by simultaneously assessing a large number of exposures, they can make sure that all exposure data are available in exactly the same subjects (maximising the comparability between exposure-specific estimates) and use more efficient statistical models allowing to better limit false positive signals due to confounding by co-exposures and thus in principle identify or get closer to the causal variable(s). All this suggests to advise teams who have previously published successive articles on a given outcome in a specific cohort to try to conduct an analysis simultaneously considering all the exposures independently considered previously. We have already discussed other challenges of exposome studies, such as the curse of dimension and the possibly resulting low power, as well as possible cures.

## Conclusion

In conclusion, we provided a typology of the main exposome studies illustrating their ability to cover most of the core aims of environmental health research. This view is probably more public health-oriented than a large fraction of the previous reviews on the exposome (Supplementary Table 1), which tend to position exposome research as a way to more efficiently unravel the underlying mechanisms of the health effects of environmental factors, in addition to identifying these health effects. At least in humans, we believe that unravelling such mechanisms is not made easier, and is probably more challenging in an exposome context than in single exposure studies, as untargeted mechanistic studies heavily suffer from the curses of dimensionality and of correlation and measurement error between exposures and biomarkers of effect, all the more in a multi-exposure context.

As mentioned in the introduction, epidemiologists have actually been conducting exposome-type studies well before Wild coined the term in 2005. This could be better recognized in the stages of study design and analysis. The first twenty years of exposome research demonstrated that it is possible to simultaneously (i.e. in the same study subjects) characterise many components of the exposome in relatively large populations, rather than studying its components in isolation. In spite of this impressive progress, expanding the coverage of the exposome may not be achieved as quickly as the coverage of the human genome. This is because, unlike for the genome, the exposome cannot currently be quantified relying on one single biological molecule or assessment tool; also, the exposome varies quickly within subjects, across tissues and organs, contrarily to the genome. Even rather low correlations between exposures give rise to a high false positive rate in etiologic exposome studies when applying ExWAS (exposure-wide association studies) to systematically associate many exposures to a health outcome; alternative statistical analysis strategies are available [2, 66]. Expanding the dimension of the exposome assessed should not be done at the cost of a decrease in the accuracy of estimation of each exposure, compared to single exposure studies, in particular if the aim is etiological. Contrarily to what tends to be observed in the literature, as the number of exposures simultaneously assessed grows in such etiologic studies, sample size should also increase, as well as the accuracy of the assessment of exposures. Translating the results of these etiologic studies into information meaningful for public health and public decision is possible: exposome-wide health impact assessment (or environmental disease burden) studies are feasible, provided representative biomonitoring or cohort studies are conducted to document distributions of exposures in the population, and provided strong efforts are made to synthesize the level of evidence and existing dose–response functions, allowing to increase the fraction of the exposome considered in such studies. These environmental disease burden assessments can provide a ranking of the health impact of a large number of exposures, allowing society to prioritize those warranting action. These can, finally, be targeted in intervention studies or assessment of policies, requiring to mobilize a wide range of disciplines from quantitative sciences to humanity, allowing to highlight how societies can efficiently control exposures to improve population health.

## Supplementary Information

Supplementary Material 1
