# Correction: Relevance and challenges of exposome studies for environmental health research

**DOI:** 10.1186/s12940-026-01317-4

**Published:** 2026-07-10

**Authors:** Rémy Slama, Valérie Siroux, Martine Vrijheid, Xavier Basagaña

**Affiliations:** 1 SMILE Team, Ecole Normale Supérieure, Institut de Biologie de L’ENS (IBENS), Université PSL, CNRS, Inserm, Paris, 75005 France; 2 PARSEC (Paris Research in Health, Environment and Climate), Ecole Normale Supérieure, Inserm, Paris, 75005 France; 3 Team of Environmental Epidemiology Applied to Development and Respiratory Health, Institute for Advanced Biosciences, UMR 5309, University Grenoble Alpes, CNRS, Inserm U 1209, Grenoble, France; 4 ISGlobal, Barcelona, Spain; 5 Universitat Pompeu Fabra, Barcelona, Spain; 6 Spanish Consortium for Research On Epidemiology and Public Health (CIBERESP), Madrid, Spain

**Correction: Environ Health 25, 49 (2026)**

**https://doi.org/10.1186/s12940-026-01299-3**

Following the publication of the original article [1], it was noted that due to a typesetting error an old version of figures 1 and 3A had been used in the first published version of the article. Some minor typos were also not implemented.

The correct figures have been included in this correction, and the original article has been corrected.

**Fig. 1:**
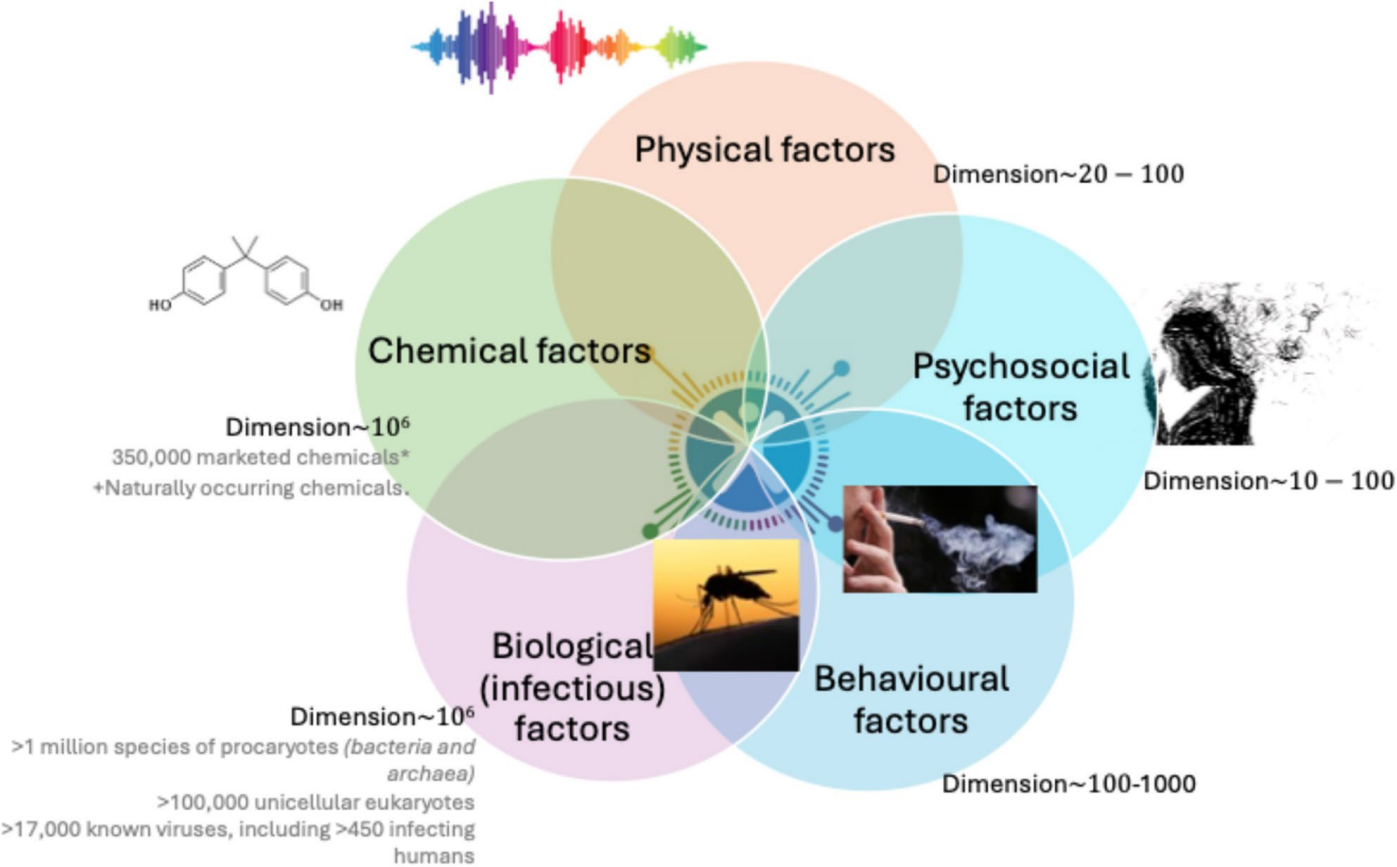
The exposome five main domains. These dimensions can be seen as encompassing lifestyle factors, aesthetic factors and systemic factors such as climate change, antimicrobial resistance or biodiversity loss (see discussion). The approximative number of exposures in each exposome domain dimen-sion comes from Wang [11] for chemicals; from Louca for bacteria and archaea [12]; from Wiens for unicellular eukaryotes (considered to correspond to the number of protist species) [13]; from He for virus [14]. See Table 3 for the remaining domains

**Fig. 3:**
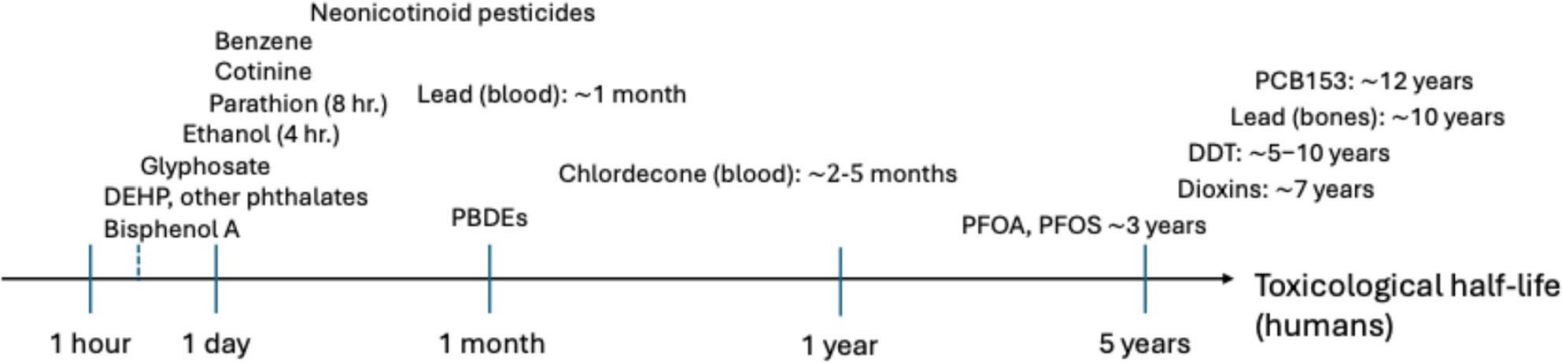
Issues related to the temporal variability in exposures. **A** Approximate half-life of select chemicals in humans. **B** Variations in urinary Bisphenol S (a chemical contaminant) concentrations in two pregnant women in whom a sample of all micturitions were collected during a week (each curve corresponds to a woman; note that concentrations vary by a ratio of about 1 to 20 in one woman and 1 to 100 in the other woman between samples collected in the same week). **C** Bias in the estimated e! ect of an exposure influencing a health outcome according to the number of biospecimens col-lected in each subject to assess exposure and to the analytical approach [47] in the case of a compound with an intra-class coe"cient of correlation of 0.2

## References

[1] Slama R, Siroux V, Vrijheid M, et al. Relevance and challenges of exposome studies for environmental health research. Environ Health. 2026;25:49. 10.1186/s12940-026-01299-3. 42210260 10.1186/s12940-026-01299-3 PMC13242124

