# Supplementary material for: Relevance and challenges of exposome studies for environmental health research
Source: Environ Health. 2026 May 29;25:49. doi: 10.1186/s12940-026-01299-3 (PMC13242124; doi:10.1186/s12940-026-01299-3)
Supplement: Supplementary file 1 — Supplementary Material 1 [file 12940_2026_1299_MOESM1_ESM.docx]

SUPPLEMENTAL MATERIAL

**Relevance and challenges of exposome studies for environmental health research**

Supplementary Table 1: Overview of reviews and commentaries on the exposome concept. Based on a literature search targeting reviews including “exposome” as a keyword.

| **Publication** | **Key content** |
| --- | --- |
| Wild, 2005 [1] | Seminal article presenting the need to develop exposome concept and research. |
| Rappaport, 2011 [2] | The exposome seen as the biologically active chemicals in the internal environment. Relevance of a “top-down” approach based upon molecules identified from human biomonitoring studies. Need for integration of research communities fractured along lines related to specific diseases and exposures. |
| Buck Louis, 2012 [3] | Challenges that exposome research needs to overcome. |
| Wild, 2012 [4] | Categorizing the exposome as including internal, specific external and general external components. Approaches to measure the exposome. Exposome research as a way to gain insight on disease mechanisms. |
| Wild, 2013 [5] | Application of omics technologies for exposome research. |
| Miller, 2014 [6] | Offers a refined definition of the exposome as “The cumulative measure of environmental influences and associated biological responses throughout the lifespan, including exposures from the environment, diet, behaviour, and endogenous processes” |
| Nakamura, 2014 [7] | Endogenous exposome, understood as DNA damage arising from the production of endogenous electrophilic molecules in the cells. |
| Rappaport, 2014 [8] | The blood exposome: description of levels, sources and biological effects of small molecules in human blood. |
| Robinson, 2015 [9] | The pregnancy exposome: review of studies characterizing the pregnancy exposome. |
| Slama, 2015 [10] | Statistical tools for exposome research and issues related to exposure assessment. |
| Jones, 2016 [11] | Review of high-resolution metabolomics (HRM) to assess environmental chemicals and biologic responses. Need to complement human studies with toxicological approaches. |
| Siroux, 2016 [12] | Challenges of exposome research in terms of exposure assessment and statistical analysis. |
| Buck Louis, 2017 [13] | Relevance of the exposome for DOHaD (Developmental origins of health and diseases) concept. Challenges of exposome research in terms of study design, analysis, exposure assessment, study outcome… |
| Escher, 2017 [14] | Relevance to integrate the exposome concept with adverse outcome pathways (AOPs), as a way to improve the understanding of mechanisms of the effects of exposures and identify the main risk drivers in complex mixtures. |
| Haddad, 2019 [15] | Characteristics of published human exposome studies (82 studies): design, exposures, health outcomes, omics tools, statistical analysis. |
| Loh, 2017 [16] | Relevance of mobile technology, sensors and the “internet of things” for exposure assessment. |
| Niedzwiecki, 2019 [17] | Relevance of the exposome concept for pharmacology and toxicology. The exposome paradigm as a way to provide data on biological responses to exposures. |
| Bocato, 2019 [18] | The interface between human biomonitoring and exposome research. |
| Santos, 2020 [19] | Statistical approaches and methodological choices when analysing exposome data, mostly in the context of birth cohorts |
| Vineis, 2020 [20] | Integration of *Omic* markers in epidemiological studies |
| Vermeulen, 2020 [21] | Relevance of high-resolution mass spectrometry and network science for exposome research. Need to estimate an exposome risk score (ERS), akin to polygenic risk scores (PRS). The exposome and regulation. Need to scale up exposome research to increase sample size of exposome studies. |
| Fang, 2021 [22] | Exposome studies should focus on critical windows of exposure, rely on data- and hypothesis-driven approaches and use reliable statistical analysis tools for high-dimensional data. |
| Zhang P, 2021 [23] | Acknowledgment of the fact that exposome research expanded to include the biological response to environmental exposures. Presentation of top-down and bottom-up exposome approaches. Use of Mass spectrometry to assess exposures. Role of exposome in precision medicine. A call to create a comprehensive exposome (exposures) database. |
| Zhang X, 2021 [24] | Connection between quantified self/self-tracking approaches and exposome research. |
| Barouki, 2022 [25] | Interfacing the exposome framework and contemporary mechanistic toxicology. Relevance of adverse outcome pathways (AOPs) and aggregate exposure pathways (AEPs). Eco-exposome concept. |
| Deguen, 2022 [26] | Document how socio-economic status could modify the association between environmental nuisances and health outcomes, across different ages. |
| Vineis, 2022 [27] | The exposome as a unifying concept for epidemiology and toxicology. Social differences, inequalities and more generally the *social capital* (Bourdieu) as influencing downstream layers. Need to consider life-course adverse outcome pathways as a way to integrate AOPs in exposome research. |
| Wei, 2022 [28] | Summary of the biological and chemical exposomes in air, soil and water, and their mutual interactions as well as with the physical exposome. |
| Karlsson, 2023 [29] | Review of the central role of mechanistic toxicology in chemical safety assessment. |
| Merra, 2024 [30] | Exposome-microbiome relationship. |
| Miller, 2025 [31] | Defining the field of *exposomics* as examining the comprehensive and cumulative effects of all physical, chemical, biological and psychosocial factors by integrating data from interdisciplinary methodologies and data streams. Exposomics distinguished from environmental health research “in that it involves a longitudinal assessment across multiple environmental domains, ultimately linking them with effects on health”. Need for methods interrogating exposure mechanisms and cascading biological responses. |
| Petit, 2025 [32] | Bibliometric analysis of exposome studies |
| Barouki, 2023 [33]; Barzilay, 2022 [34] ; Danieli, 2024 [35]; Dréno, 2018; Goutman, 2023 [36]; Ibanez, 2024 [37]; Motairek, 2023; Muenzel, 2023 [38]; Young, 2025 [39] | Examples of reviews on the relevance of the exposome or its contribution to specific diseases (*not detailed**) |

*Reviews on disease-specific topics (such as the influence of exposome on acne) have not been systematically listed.

AOP: Adverse outcome pathways.

REFERENCES

1. Wild CP. Complementing the Genome with an “Exposome”: The Outstanding Challenge of Environmental Exposure Measurement in Molecular Epidemiology. Cancer Epidemiol Biomarkers Prev [Internet]. 2005 [cited 2021 July 14];14:1847–50. https://doi.org/10.1158/1055-9965.EPI-05-0456

2. Rappaport SM. Implications of the exposome for exposure science. J Expo Sci Environ Epidemiol [Internet]. 2011 [cited 2026 Mar 13];21:5–9. https://doi.org/10.1038/jes.2010.50

3. Buck Louis GM, Sundaram R. Exposome: time for transformative research. Stat Med [Internet]. 2012 [cited 2026 Mar 16];31:2569–75. https://doi.org/10.1002/sim.5496

4. Wild CP. The exposome: from concept to utility. Int J Epidemiol [Internet]. 2012 [cited 2026 Mar 13];41:24–32. https://doi.org/10.1093/ije/dyr236

5. Wild CP, Scalbert A, Herceg Z. Measuring the exposome: A powerful basis for evaluating environmental exposures and cancer risk. Environ Mol Mutagen [Internet]. 2013 [cited 2026 Mar 16];54:480–99. https://doi.org/10.1002/em.21777

6. Miller GW, Jones DP. The Nature of Nurture: Refining the Definition of the Exposome. Toxicol Sci [Internet]. 2014 [cited 2025 Nov 21];137:1–2. https://doi.org/10.1093/toxsci/kft251

7. Nakamura J, Mutlu E, Sharma V, Collins L, Bodnar W, Yu R, et al. The endogenous exposome. DNA Repair [Internet]. 2014 [cited 2026 Mar 13];19:3–13. https://doi.org/10.1016/j.dnarep.2014.03.031

8. Rappaport SM, Barupal DK, Wishart D, Vineis P, Scalbert A. The Blood Exposome and Its Role in Discovering Causes of Disease. Environ Health Perspect [Internet]. 2014 [cited 2026 Mar 13];122:769–74. https://doi.org/10.1289/ehp.1308015

9. Robinson O, Vrijheid M. The Pregnancy Exposome. Curr Environ Health Rep [Internet]. 2015 [cited 2026 Mar 13];2:204–13. https://doi.org/10.1007/s40572-015-0043-2

10. Slama R, Vrijheid M. Some challenges of studies aiming to relate the Exposome to human health. Occup Environ Med [Internet]. 2015 [cited 2026 Mar 13];72:383–4. https://doi.org/10.1136/oemed-2014-102546

11. Jones DP. Sequencing the exposome: A call to action. Toxicol Rep [Internet]. 2016 [cited 2026 Mar 13];3:29–45. https://doi.org/10.1016/j.toxrep.2015.11.009

12. Siroux V, Agier L, Slama R. The exposome concept: a challenge and a potential driver for environmental health research. Eur Respir Rev [Internet]. 2016 [cited 2021 July 15];25:124–9. https://doi.org/10.1183/16000617.0034-2016

13. Buck Louis GM, Smarr MM, Patel CJ. The Exposome Research Paradigm: an Opportunity to Understand the Environmental Basis for Human Health and Disease. Curr Environ Health Rep [Internet]. 2017 [cited 2026 Mar 16];4:89–98. https://doi.org/10.1007/s40572-017-0126-3

14. Escher BI, Hackermüller J, Polte T, Scholz S, Aigner A, Altenburger R, et al. From the exposome to mechanistic understanding of chemical-induced adverse effects. Environ Int [Internet]. 2017 [cited 2026 Mar 13];99:97–106. https://doi.org/10.1016/j.envint.2016.11.029

15. Haddad N, Andrianou XD, Makris KC. A Scoping Review on the Characteristics of Human Exposome Studies. Curr Pollut Rep [Internet]. 2019 [cited 2026 Mar 16];5:378–93. https://doi.org/10.1007/s40726-019-00130-7

16. Loh M, Sarigiannis D, Gotti A, Karakitsios S, Pronk A, Kuijpers E, et al. How Sensors Might Help Define the External Exposome. Int J Environ Res Public Health [Internet]. 2017 [cited 2026 Mar 16];14:434. https://doi.org/10.3390/ijerph14040434

17. Niedzwiecki MM, Walker DI, Vermeulen R, Chadeau-Hyam M, Jones DP, Miller GW. The Exposome: Molecules to Populations. Annu Rev Pharmacol Toxicol [Internet]. 2019 [cited 2026 Mar 13];59:107–27. https://doi.org/10.1146/annurev-pharmtox-010818-021315

18. Bocato MZ, Bianchi Ximenez JP, Hoffmann C, Barbosa F. An overview of the current progress, challenges, and prospects of human biomonitoring and exposome studies. J Toxicol Environ Health Part B [Internet]. 2019 [cited 2026 Mar 16];22:131–56. https://doi.org/10.1080/10937404.2019.1661588

19. Santos S, Maitre L, Warembourg C, Agier L, Richiardi L, Basagaña X, et al. Applying the exposome concept in birth cohort research: a review of statistical approaches. Eur J Epidemiol [Internet]. 2020 [cited 2021 Jan 29];35:193–204. https://doi.org/10.1007/s10654-020-00625-4

20. Vineis P, Robinson O, Chadeau-Hyam M, Dehghan A, Mudway I, Dagnino S. What is new in the exposome? Environ Int [Internet]. 2020 [cited 2026 Mar 13];143:105887. https://doi.org/10.1016/j.envint.2020.105887

21. Vermeulen R, Schymanski EL, Barabási A-L, Miller GW. The exposome and health: Where chemistry meets biology. Science [Internet]. 2020 [cited 2024 Sept 19];367:392–6. https://doi.org/10.1126/science.aay3164

22. Fang M, Hu L, Chen D, Guo Y, Liu J, Lan C, et al. Exposome in human health: Utopia or wonderland? The Innovation [Internet]. 2021 [cited 2026 Mar 16];2:100172. https://doi.org/10.1016/j.xinn.2021.100172

23. Zhang P, Carlsten C, Chaleckis R, Hanhineva K, Huang M, Isobe T, et al. Defining the Scope of Exposome Studies and Research Needs from a Multidisciplinary Perspective. Environ Sci Technol Lett [Internet]. 2021 [cited 2026 Mar 16];8:839–52. https://doi.org/10.1021/acs.estlett.1c00648

24. Zhang X, Gao P, Snyder MP. The Exposome in the Era of the Quantified Self. Annu Rev Biomed Data Sci [Internet]. 2021 [cited 2026 Mar 16];4:255–77. https://doi.org/10.1146/annurev-biodatasci-012721-122807

25. Barouki R, Audouze K, Becker C, Blaha L, Coumoul X, Karakitsios S, et al. The Exposome and Toxicology: A Win–Win Collaboration. Toxicol Sci [Internet]. 2022 [cited 2026 Mar 16];186:1–11. https://doi.org/10.1093/toxsci/kfab149

26. Deguen S, Amuzu M, Simoncic V, Kihal-Talantikite W. Exposome and Social Vulnerability: An Overview of the Literature Review. Int J Environ Res Public Health [Internet]. 2022 [cited 2026 Mar 16];19:3534. https://doi.org/10.3390/ijerph19063534

27. Vineis P, Barouki R. The exposome as the science of social-to-biological transitions. Environ Int [Internet]. 2022 [cited 2026 Mar 13];165:107312. https://doi.org/10.1016/j.envint.2022.107312

28. Wei X, Huang Z, Jiang L, Li Y, Zhang X, Leng Y, et al. Charting the landscape of the environmental exposome. iMeta [Internet]. 2022 [cited 2026 Mar 16];1:e50. https://doi.org/10.1002/imt2.50

29. Karlsson O. Chemical safety and the exposome. Emerg Contam [Internet]. 2023 [cited 2026 Mar 16];9:100225. https://doi.org/10.1016/j.emcon.2023.100225

30. Merra G, Gualtieri P, La Placa G, Frank G, Della Morte D, De Lorenzo A, et al. The Relationship between Exposome and Microbiome. Microorganisms [Internet]. 2024 [cited 2026 Mar 13];12:1386. https://doi.org/10.3390/microorganisms12071386

31. Miller GW, Banbury Exposomics Consortium. Integrating exposomics into biomedicine. Science. 2025;388:356–8.

32. Petit P, Vuillerme N. Global research trends on the human exposome: a bibliometric analysis (2005–2024). Environ Sci Pollut Res [Internet]. 2025 [cited 2026 Mar 13];32:7808–33. https://doi.org/10.1007/s11356-025-36197-7

33. Barouki R, Samson M, Blanc EB, Colombo M, Zucman-Rossi J, Lazaridis KN, et al. The exposome and liver disease - how environmental factors affect liver health. J Hepatol [Internet]. 2023 [cited 2026 Mar 16];79:492–505. https://doi.org/10.1016/j.jhep.2023.02.034

34. Barzilay R, Pries L-K, Moore TM, Gur RE, Van Os J, Rutten BPF, et al. Exposome and Trans-syndromal Developmental Trajectories Toward Psychosis. Biol Psychiatry Glob Open Sci [Internet]. 2022 [cited 2026 Mar 16];2:197–205. https://doi.org/10.1016/j.bpsgos.2022.05.001

35. Danieli MG, Casciaro M, Paladini A, Bartolucci M, Sordoni M, Shoenfeld Y, et al. Exposome: Epigenetics and autoimmune diseases. Autoimmun Rev [Internet]. 2024 [cited 2026 Mar 13];23:103584. https://doi.org/10.1016/j.autrev.2024.103584

36. Goutman SA, Savelieff MG, Jang D-G, Hur J, Feldman EL. The amyotrophic lateral sclerosis exposome: recent advances and future directions. Nat Rev Neurol [Internet]. 2023 [cited 2026 Mar 13];19:617–34. https://doi.org/10.1038/s41582-023-00867-2

37. Ibanez A, Melloni L, Świeboda P, Hynes W, Ikiz B, Ayadi R, et al. Neuroecological links of the exposome and One Health. Neuron [Internet]. 2024 [cited 2026 Mar 13];112:1905–10. https://doi.org/10.1016/j.neuron.2024.04.016

38. Münzel T, Sørensen M, Hahad O, Nieuwenhuijsen M, Daiber A. The contribution of the exposome to the burden of cardiovascular disease. Nat Rev Cardiol [Internet]. 2023 [cited 2026 Mar 13];20:651–69. https://doi.org/10.1038/s41569-023-00873-3

39. Young AS, Mullins CE, Sehgal N, Vermeulen RCH, Kolijn PM, Vlaanderen J, et al. The need for a cancer exposome atlas: a scoping review. JNCI Cancer Spectr [Internet]. 2025 [cited 2026 Apr 1];9:pkae122. https://doi.org/10.1093/jncics/pkae122
